# The Jahn-Teller Effect for Amorphization of Molybdenum Trioxide towards High-Performance Fiber Supercapacitor

**DOI:** 10.34133/2021/6742715

**Published:** 2021-03-29

**Authors:** Chenyang Yu, Hai Xu, Yujiao Gong, Ruyi Chen, Zengyu Hui, Xi Zhao, Yue Sun, Qiang Chen, Jinyuan Zhou, Wenxin Ji, Gengzhi Sun, Wei Huang

**Affiliations:** ^1^Institute of Advanced Materials (IAM), Nanjing Tech University (NanjingTech), Nanjing 211816, China; ^2^School of Materials Science and Engineering, Henan Polytechnic University, Jiaozuo 454003, China; ^3^School of Physical Science and Technology, Lanzhou University, Lanzhou 730000, China; ^4^State Key Laboratory of High-Efficiency Coal Utilization and Green Chemical Engineering, Ningxia University, Yinchuan 750021, China; ^5^Institute of Flexible Electronics (IFE), Northwestern Polytechnical University (NPU), Xi'an 710072, China

## Abstract

Amorphous pseudocapacitive nanomaterials are highly desired in energy storage applications for their disordered crystal structures, fast electrochemical dynamics, and outstanding cyclic stability, yet hardly achievable using the state-of-the-art synthetic strategies. Herein, for the first time, high capacitive fiber electrodes embedded with nanosized amorphous molybdenum trioxide (A-MoO_3-x_) featuring an average particle diameter of ~20 nm and rich oxygen vacancies are obtained *via* a top-down method using *α*-MoO_3_ bulk belts as the precursors. The Jahn-Teller distortion in MoO_6_ octahedra due to the doubly degenerate ground state of Mo^5+^, which can be continuously strengthened by oxygen vacancies, triggers the phase transformation of *α*-MoO_3_ bulk belts (up to 30 *μ*m long and 500 nm wide). The optimized fibrous electrode exhibits among the highest volumetric performance with a specific capacitance (*C*_*V*_) of 921.5 F cm^−3^ under 0.3 A cm^−3^, endowing the fiber-based weaveable supercapacitor superior *C*_*V*_ and *E*_*V*_ (energy density) of 107.0 F cm^−3^ and 9.5 mWh cm^−3^, respectively, together with excellent cyclic stability, mechanical robustness, and rate capability. This work demonstrates a promising strategy for synthesizing nanosized amorphous materials in a scalable, cost-effective, and controllable manner.

## 1. Introduction

Wearable devices, ranging from epidermal electronic skins [1, 2], bendable displays [3, 4], weaveable memories [5], smart textiles [6–8], and healthcare sensors [9, 10] to flexible solar cells [11, 12] and nanogenerators [13, 14], are leading a new electronic revolution and desperately demand for matchable energy storage devices with superior volumetric capacitance that can store and supply power efficiently. Among all candidates, fiber supercapacitors (FSCs) are of particular interest owing to their good flexibility, lightweight, high wearing safety, and long servicing lifespan [15–18]. Many efforts have been paid to enhance the energy performance of FSCs by incorporating pseudocapacitive nanomaterials, such as vanadium oxide (V_2_O_5_), manganese oxide (MnO_2_), and molybdenum oxide (MoO_3_), due to their high theoretical capacitances originated from the multivalent redox centers [19–23]. Nevertheless, the aforementioned materials suffer from sluggish ion/electron transportation because of their highly crystalized structure with anisotropic characteristics and wide bandgaps. In addition, the distinct volume expansion during charge-discharge (ion deintercalation-intercalation) results in rapid performance decay. Therefore, the exploration of advanced electrode materials with efficient charge storage capability is highly required.

As a typical transitional metal oxide, MoO_3_ possess high theoretical specific capacitance due to the multielectron transfer process during proton intercalation-deintercalation [24, 25]. Compared with crystalline counterparts, nanosized amorphous MoO_3_ (A-MoO_3_) are highly desired for their disordered structures, good chemical stability, high elasticity, low internal energy, and large surface area, enabling isotropic ion-diffusion, enhanced electrochemical dynamics, excellent mechanical robustness, and superior structure stability during charge/discharge cycling [26]. Ideally, the synthesis of A-MoO_3_ should be convenient with tunable compositions (e.g., oxygen vacancies and hybridization), free of contamination (e.g., surfactants) for eliminating possible blockage of active sites, and scalable for industrial production. However, the currently utilized bottom-up strategies, for example, solvothermal reaction [27, 28], electrochemical deposition [26], and photochemical deposition [29], are subjected to numerous issues, including (i) special attention needs be paid to reaction environment (e.g., temperature) to stabilize the metastable amorphous phase, (ii) nonuniform coating resulted from unevenly distributed electric field on electrode surface, (iii) rigorous experimental conditions, e.g., ultraviolet illumination, (iv) possible contamination from organic additives, and (v) complicated procedures which are not suitable for massive preparation. Moreover, these methods are incompatible with the state-of-the-art fiber-spinning techniques [30, 31].

Herein, for the first time, high-performance fiber electrodes embedded with nanosized A-MoO_3-x_ featuring an average particle diameter of 20 nm and rich oxygen vacancies are obtained *via* a newly developed top-down strategy by directly phase transformation of *α*-MoO_3_ crystals. The underlying mechanism for amorphization and nanocrystallization is highlighted and attributed to the strengthened Jahn-Teller distortion in MoO_6_ octahedra arising from the doubly degenerate ground state of Mo^5+^. The enlarged interfacial contact between A-MoO_3-x_ and rGO endues the obtained A-MoO_3-x_/rGO hybrid fiber an ultrahigh *C*_*V*_ of 921.5 F cm^−3^ under 0.3 A cm^−3^ in 1 M H_2_SO_4_, enabling the assembled solid-state FSC superior *C*_*V*_ and *E*_*V*_ of 107.0 F cm^−3^ and 9.51 mWh cm^−3^, respectively, together with outstanding flexibility, cyclic stability, and rate capability.

## 2. Results

**Figure 1:**
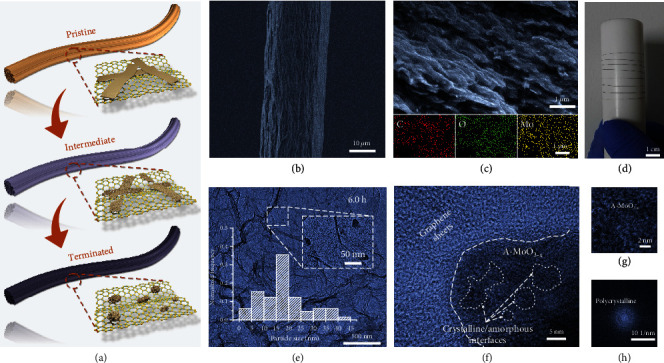
Synthesis and characterization of A-MoO_3-x_/rGO hybrid fiber. (a) Schematic illustration showing the structure evolution of *α*-MoO_3_ belts during the preparation of A-MoO_3-x_/rGO hybrid fibers. (b) SEM (scanning electron microscope) image of A-MoO_3-x_/rGO hybrid fiber obtained at 6.0 h. (c) Cross-sectional SEM image of the obtained A-MoO_3-x_/rGO hybrid fiber and the corresponding EDS mapping. (d) Digital photograph of A-MoO_3-x_/rGO hybrid fiber wounded on a PTFE rod. (e) TEM (transmission electron microscope) image of A-MoO_3-x_/rGO hybrid fiber at the synthetic time of 6.0 h. The insets are particle size distribution of A-MoO_3-x_ on rGO nanosheets and the local TEM image with higher magnification, respectively. (f–h) HRTEM and the corresponding FFT images of A-MoO_3-x_/rGO hybrid fiber obtained at 6.0 h.

When the degenerate orbitals in transition metal-based octahedral complexes are occupied by an odd number of electrons, the Jahn-Teller effect occurs through crystal distortion in order to lower the overall energy. Herein, the Jahn-Teller effect was employed for phase engineering of *α*-MoO_3_ simply triggered by oxygen vacancies. *α*-MoO_3_ belts with dimensional features in micron-scale (Figure S1) were synthesized *via* hydrothermal reaction between Mo powder and hydrogen peroxide at 220°C for 15.0 h. The as-synthesized MoO_3_ possess well-defined belt-like morphology with a length of 5 to 30 *μ*m and a width of 200 to 500 nm. The lattice spacings of ~0.36 nm and ~0.39 nm shown in Figure S2 are well indexed to (001) and (100) planes of *α*-MoO_3_ [32]. The characteristic peaks in X-ray diffraction (XRD) pattern (Figure S3a) match those of *α*-MoO_3_ crystals with orthorhombic phase well (JCPDs: 05-0508). In a typical experiment schematically illustrated in Figure 1(a), *α*-MoO_3_ belts were hydrothermally reacted with graphene oxide (GO) nanosheets in sealed glass capillaries (~1 mm in diameter) at 160°C for 6.0 h to achieve nanosized A-MoO_3-x_ and simultaneously obtain A-MoO_3-x_/rGO hybrid fibers. As indicated in Figure 1(b), the resultant A-MoO_3-x_/rGO hybrid fiber possesses a wrinkle surface with a diameter of ~30.1 *μ*m. Although no observable particles can be found under the cross-section view of A-MoO_3-x_/rGO hybrid fiber (Figure 1(c)), C, O, and Mo are actually distributed uniformly, which was evidenced by the energy dispersive spectrometer (EDS). The fiber length was facilely adjusted by the size of a microreactor, and the obtained long A-MoO_3-x_/rGO hybrid fiber (Figure 1(d)) could be bent and wound on a PTFE rod, demonstrating its good flexibility. The resultant A-MoO_3_ nanoparticles (~20 nm in diameter) are evenly anchored on rGO nanosheets (Figure 1(e)), and the amorphous phase is formed with very small crystalline domains inside not completely transformed (Figures 1(f)–1(h)). The closed diffraction rings with several highlight points in the FFT pattern (Figure 1(h)) also suggest the existence of polycrystalline structure in the nanoparticles. This is in good accordance with the XRD pattern (Figure S3b), in which the characteristic peaks of *α*-MoO_3_ crystals disappear after hydrothermal treatment and the broadened peak is attributable to rGO. By contrast, the characteristic XRD pattern of the orthonormal phase maintains when *α*-MoO_3_ belts were hydrothermally treated without any additives or with graphite powder (Figure S4), indicating that GO is crucial for the amorphization and collapse of *α*-MoO_3_ crystals.

**Figure 2:**
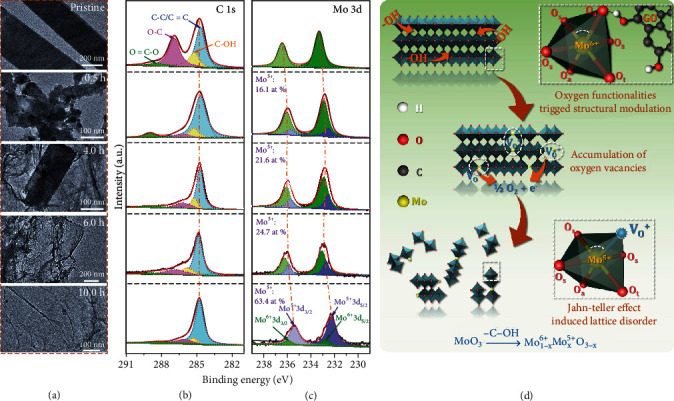
Structure evolution of *α*-MoO_3_ belts during hydrothermal treatment. (a) TEM images of *α*-MoO_3_ belts and A-MoO_3-x_/rGO hybrid fibers obtained at different synthetic time. XPS spectra of (b) C 1s and (c) Mo 3d in pristine GO, bare *α*-MoO_3_ belts, and hybrid fibers (A-MoO_3-x_/rGO) obtained at different synthetic time. (d) The Jahn-Teller distortion during the reaction between *α*-MoO_3_ and oxygen functionalities on GO.

Ex situ TEM images clearly reflect the morphology evolution of *α*-MoO_3_ belts during hydrothermal reaction with GO from 0.5 to 10.0 h (Figure 2(a)), where the pristine *α*-MoO_3_ belts with large size were collapsed into small particles at 0.5 h and fully converted to nanosized A-MoO_3-x_ at 6.0 h. The size and phase transformation of *α*-MoO_3_ belts can also be evidenced from the SEM images and XRD patterns of intermediate state A-MoO_3-x_/rGO hybrid fibers (Figures S5 and S6). Furthermore, the chemical states and valance evolution of C and Mo were investigated by X-ray photoelectron spectroscopy (XPS). The four deconvoluted peaks of C 1s can be well ascribed to C-C (284.6 eV), C-OH (285.2 eV), C-O (286.9 eV), and O=C-OH (288.5 eV) on GO nanosheets, respectively, suggesting the existence of abundant oxygen-containing groups (Figure 2(b)). As expected, upon hydrothermal treatment, the oxygen-containing groups were effectively eliminated from GO nanosheets, restoring the high electrical conductivity. The controllable manipulation of the concentration of oxygen vacancies is observed from the fine-scanned Mo 3d region. The characteristic peaks at 232.9 eV (Mo^6+^ 3d_5/2_) and 236.1 eV (Mo^6+^ 3d_3/2_) tend to downshift (Figure 2(c)), suggesting that oxygen vacancies are gradually generated with an increasing content of Mo^5+^ (16.1 at%, 21.6 at%, 24.7 at%, and 63.4 at% at 0.5 h, 4.0 h, 6.0 h, and 10.0 h, respectively) and *α*-MoO_3_ crystals are reduced under hydrothermal condition. The reaction process can be simplified as follows:

(1) $$\begin{array}{c} {\text{MoO}}_{3}\overset{-\text{C}-\text{OH}}{\to }{\text{Mo}}_{1-\text{x}}^{6+}{\text{Mo}}_{\text{x}}^{5+}{\text{O}}_{3-\text{x}} \end{array}$$

**Figure 3:**
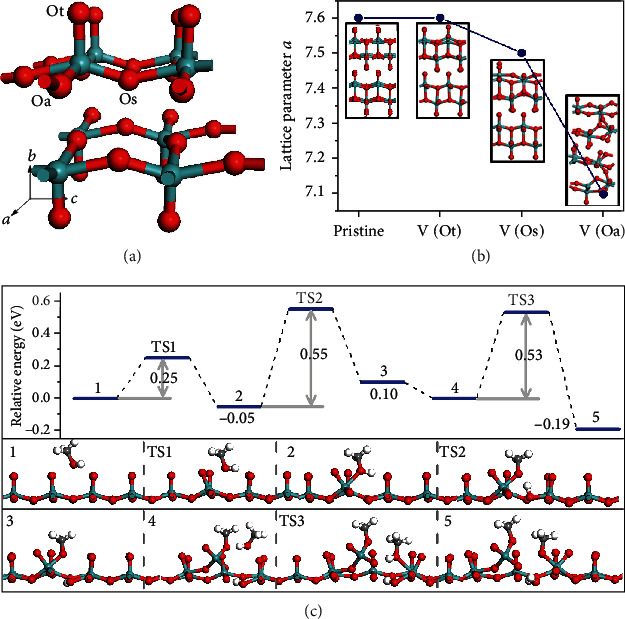
DFT calculation showing the lattice distortion of *α*-MoO_3_. (a) Three types of surface oxygen atoms in *α*-MoO_3_. (b) Dependence of lattice parameter change on the type of oxygen vacancy. (c) Reaction energy profile of vacancy formation (top) and the corresponding transition states and intermediates (bottom).

The overall amorphization and collapse of *α*-MoO_3_ belts can be ascribed to the strengthened Jahn-Teller distortion, as illustrated in Figure 2(d). According to crystal field stabilization energy of *α*-MoO_3_, lattice distortion happens in [MoO_6_] unit due to the destroyed symmetry of electron cloud, which is known as the Jahn-Teller effect [33, 34]. In addition, the electronic instability of Mo^5+^, which can be created by introducing oxygen vacancies in *α*-MoO_3_, will further enhance the Jahn-Teller distortion, leading to changes in crystal and electronic structure [21, 35]. The formation of oxygen vacancies and the deformation of surface lattice were theoretically unveiled using density functional theory (DFT), giving further evidence on the strengthened Jahn-Teller distortion. As shown in Figure 3(a), surface oxygen atoms can be classified into three types, Ot, Oa, and Os, respectively, upon the differences of the chemical state (chemical bonding or van der Waals force). For example, Ot vacancy (V(Ot)) reserves the instinct lattice symmetry with slight atomic displacement, whereas V(Os) and V(Oa) induce large lattice deformation (Figure 3(b)), e.g., 6% of lattice shrink for V(Oa) [36, 37]. Since the oxygen-containing groups, e.g., C–OH, C=O, and -COOH, generally play a critical role in interface reactions, by taking GO nanosheets into consideration, approximate models of CH_3_OH and H_2_CO were adopted to elucidate the formation of oxygen vacancies. Our calculation shows that *α*-MoO_3_ has a strong affinity towards C-OH groups rather than C=O, and the energy barrier for the linkage between Mo atom and -OH is 0.25 eV (Figure 3(c)). Based on Bader charge analysis, Oa and Os are more negatively charged than Ot; consequently, H atoms are prone to contact with Oa and Os, forming an intermediate of ∗Oa-H (or Os-H) with an energy barrier of 0.55 eV. Subsequently, when a second protonation process occurs on the surface, an oxygen vacancy is generated together with a corresponding Mo^5+^ Jahn-Teller center, which would destabilize the octahedral environments *via* stretching and contraction Mo-O bonds and boost more Oa and Os site exploration [38–40]. Therefore, in our experiment, the abundant oxygen-containing functional groups (e.g., C-OH, C=O, and C-O-C as shown in Figure 2(b)) on GO nanosheets hydrothermally reacted with *α*-MoO_3_ at 160°C. The accumulation of oxygen vacancies led to the rupture of Mo-O bonds in MoO_6_ octahedra and the deformation of surface lattice. Ultimately, the amorphization and collapse of *α*-MoO_3_ into amorphous nanoparticles were achieved.

**Figure 4:**
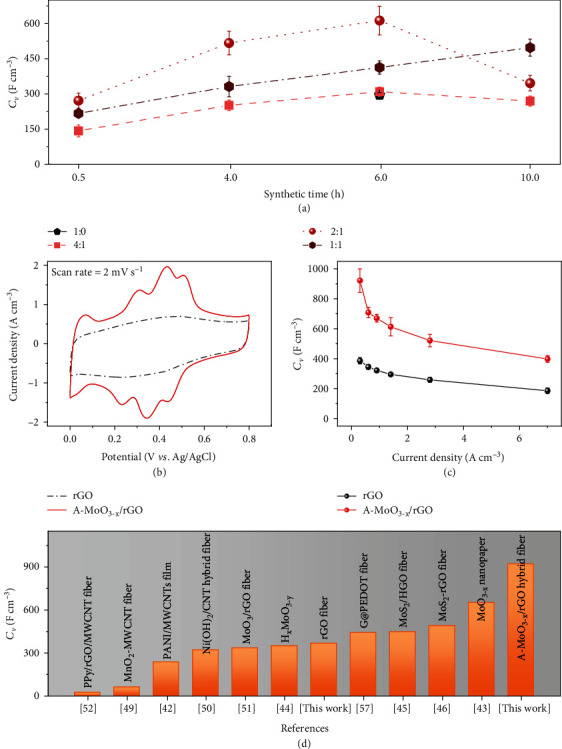
Electrochemical performances of A-MoO_3-x_/rGO hybrid fiber electrodes. (a) *C*_*V*_ of A-MoO_3-x_/rGO hybrid fiber electrodes versus synthetic time and the feeding ratio between GO and *α*-MoO_3_ belts at 1.4 A cm^−3^. (b) CV curves of hybrid fiber and rGO fiber at 2 mV s^−1^. (c) Current density-dependent *C*_*V*_ of the hybrid fiber and rGO fiber. (d) Comparison of the highest *C*_*V*_ based on a single flexible electrode in this work and previous studies.

Guided by the above mechanism, the electrochemical performance of the A-MoO_3-x_/rGO fiber electrode was optimized *via* simply regulating the time of hydrothermal treatment and feeding ratio of GO and *α*-MoO_3_. The cyclic voltammograms (CVs) at the scan rate of 2 mV s^−1^ were measured in a three-electrode configuration within the potential window between 0 and 0.8 V using Ag/AgCl (saturated KCl) as the reference electrode and H_2_SO_4_ (1 M) as the acidic electrolyte, respectively. As expected, moderate amount of oxygen vacancies, uniform loading of amorphous nanoparticles, and the mild reduction of GO nanosheets together endow the resultant A-MoO_3-x_/rGO fiber the maximum current density in CV curves at 2 mV s^−1^ and longest discharge time in GCD (galvanostatic charge-discharge) profiles at 1.4 A cm^−3^ (Figure S7), respectively. The *C*_*V*_ calculated from GCD results measured at 1.4 A cm^−3^ peaks at 612.7 F cm^−3^ (corresponding to 1.33 F cm^−2^ at 3.1 mA cm^−2^) for A-MoO_3-x_/rGO fiber obtained at 6.0 h hydrothermal reaction with the feeding ratio of 2 : 1 between GO and *α*-MoO_3_ (Figure 4(a)). It is noteworthy that the extended hydrothermal reaction time (10.0 h) leads to degradation of fiber performance for the feeding ratio of 4 : 1 and 2 : 1 probably attributable to the extensive elimination of the oxygen-containing groups on GO nanosheets, whereas the continuous capacitance increase for 1 : 1 samples can be ascribed to the incomplete conversion of *α*-MoO_3_. This observation adheres to the above-proposed mechanism in Figure 2(d). Compared to the approximate rectangular-shaped CV curve of bare rGO fiber, the A-MoO_3-x_/rGO fiber electrode exhibits much larger current density with three pairs of stable and prominent redox peaks which can be ascribed to the multielectron transfer process of the Mo centers (Figure 4(b)) [41]. By contrast, pristine *α*-MoO_3_ belts show rapid capacitance decay during cycling (Figure S8). The optimized A-MoO_3-x_/rGO fiber electrode delivered an ultrahigh *C*_*V*_ of 921.5 F cm^−3^ at 0.3 A cm^−3^, retaining 397.8 F cm^−3^ at 7.0 A cm^−3^ (Figures 4(c) and S9). This performance is 2.5-fold improvement compared to that of bare rGO electrode and much superior to those of PANI/MWCNT film (238 F cm^−3^) [42], MoO_3-x_ nanopaper (652 F cm^−3^) [43], H_x_MoO_3−y_ (350 F cm^−3^) [44], MoS_2_/HGO fiber (448 F cm^−3^) [45], and MoS_2_-rGO fiber (491.1 F cm^−3^) [46] reported in literatures (Figure 4(d)). The electrochemical behavior of A-MoO_3-x_/rGO fiber electrode was further characterized using electrochemical impedance spectroscopy (EIS). The EIS slope of hybrid fiber electrode at low-frequency range is slightly lower than that of bare rGO fiber electrode, indicating compromised ion diffusion for hybrid fibers (Figure S10). In addition, by employing Dunn's approach [47, 48], the diffusion-contributed and capacitive-contributed capacitances of A-MoO_3-x_/rGO hybrid fiber were calculated as illustrated in Figure S11. The result shows that at 2 mV s^−1^, the hybrid fiber exhibits a *C*_*V*_ of ~732.0 F cm^−3^ with 50.6% capacitive contribution, attributable to the shortened ion diffusion pathway and improved electron transport conductivity of A-MoO_3-x_ nanoparticles compared to *α*-MoO_3_ crystals (Figure S12).

**Figure 5:**
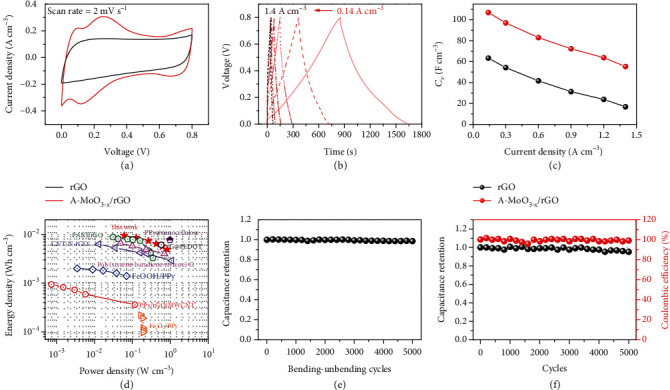
Electrochemical properties of assembled FSCs. (a) CV curves of flexible supercapacitors assembled by A-MoO_3-x_/rGO hybrid fibers and bare rGO fibers at 2 mV s^−1^. (b) GCD curves of FSC made of A-MoO_3-x_/rGO hybrid fibers and bare rGO fibers. (c) *C*_*V*_ comparison of FSCs made of A-MoO_3-x_/rGO hybrid fibers and bare rGO fibers, respectively. (d) Ragone plots of FSC made from A-MoO_3-x_/rGO hybrid fibers compared with other flexible supercapacitors. (e) Bending-unbending and (f) cycling performance of FSC made of A-MoO_3-x_/rGO hybrid fibers at 2.0 A cm^−3^.

By, respectively, using A-MoO_3-x_/rGO and rGO fibers as electrodes, symmetric FSCs were assembled. CV profiles in H_2_SO_4_/PVA gel electrolyte were obtained under 2 mV s^−1^. The FSC based on rGO fiber exhibits an almost rectangular CV curve, indicating a typical EDLC behavior. Comparably, A-MoO_3-x_/rGO FSC (Figure 5(a)) delivers a larger current density with a pair of obvious peaks corresponding to the redox chemistry on A-MoO_3-x_. According to the GCD profiles in Figure 5(b), the A-MoO_3-x_/rGO FSC exhibits an ultrahigh *C*_*V*_ of 107.0 F cm^−3^ (corresponding to 128.6 mF cm^−2^, 3.94 mF cm^−1^, and 78.3 F g^−1^, respectively) at 0.14 A cm^−3^ (Figure 5(c)), which stands at a high level compared to that of other flexible supercapacitors, e.g., 10.9 F cm^−3^ for MnO_2_-MWCNT fiber device (0.1 A cm^−3^), 28.1 F cm^−3^ for Ni(OH)_2_/CNT fiber device (0.4 A cm^−3^), and 53.5 F cm^−3^ for MoO_3_/rGO-based asymmetric device (0.1 A cm^−3^) [49–51]. At 1.4 A cm^−3^, the capacitance decreases to 55.3 F cm^−3^ with 51.7% retention. In contrast, only 26.9% of the initial capacitance (63.1 F cm^−3^) was retained for the rGO-based device when the current density increased from 0.14 to 1.4 A cm^−3^. According to the equations of *E*_*V*_ = 1/2 × *C*_*V*_ × *V*^2^ and *P*_*V*_ = *E*_*V*_/*t*_discharge_ (*E*_*V*_ and *P*_*V*_, respectively, represent the volumetric energy density and volumetric power density, *V* is the working voltage, while *t*_discharge_ stands for the discharge time), *E*_*V*_ of the A-MoO_3-x_/rGO FSC increases from 4.5 to 9.5 mWh cm^−3^ with the corresponding *P*_*V*_ varying between 0.84 and 0.06 W cm^−3^. As shown in Figure 5(d), the performance of our device is superior to the flexible supercapacitors based on PPy/rGO/MWCNT (0.94 mWh cm^−3^) [52], Fe_2_O_3_/PPy (0.22 mWh cm^−3^) [53], FeOOH/PPy (2.0 mWh cm^−3^) [54], CNT/N-rGO (6.3 mWh cm^−3^) [55], and poly(styrene-butadiene-styrene)-G (6.6 mWh cm^−3^) [56] and competitive to those based on G@PEDOT (7.0 mWh cm^−3^) [57], PPy@nanocellulose (7.7 mWh cm^−3^) [58], and PANI/rGO (8.8 mWh cm^−3^) [59]. In addition, the A-MoO_3-x_/rGO FSC exhibits excellent flexibility and cycling stability. 98.6% capacitance was sustained after bending-unbending for 5000 cycles (Figure 5(e)), and 90.4% capacitance was retained after GCD cycling for 5000 times at 2.0 A cm^−3^ (Figure 5(f)).

**Figure 6:**
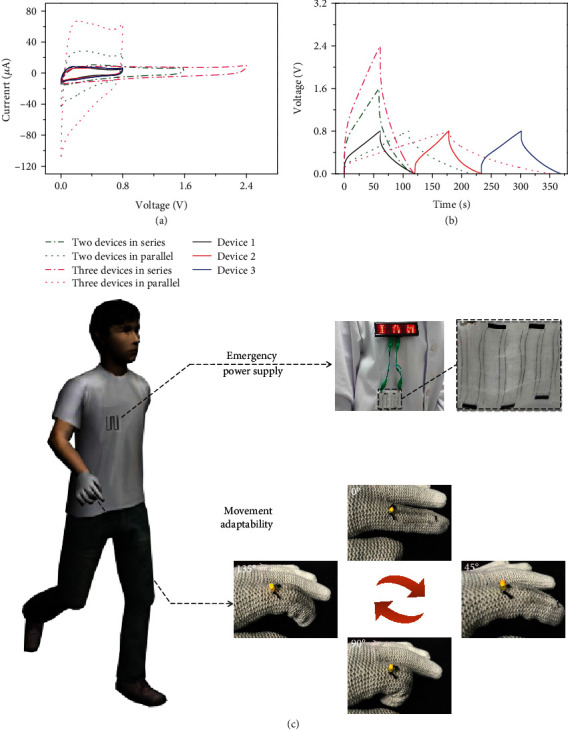
Practical demonstrations of FSCs made of A-MoO_3-x_/rGO hybrid fibers. (a) CV curves at 5 mV s^−1^ and (b) GCD profiles at 0.9 A cm^−3^ of one, two, and three FSCs connected in series and in parallel. (c) Practical demonstration of flexible FSC tandems (containing 6 and 3 devices, respectively) for lightening LED pattern of “IAM” and a yellow LED mounted on a glove.

To satisfy specific demands on energy and power, supercapacitors are required to be assembled in series or in parallel. Three hybrid fiber-based supercapacitors presenting similar CV and GCD behaviors were picked out. As shown in Figures 6(a) and 6(b), respectively, by connecting three devices in series, the output voltage can be extended to 3 times, while the connection in parallel tripled both the CV current and GCD discharge time. Moreover, as practical demonstration, a red LED array spelled word “IAM” could be lightened by a tandem system containing 6 FSCs on a piece of gauze (Figure 6(c)). Meanwhile, three hybrid fiber devices connected in series and braided on a cloth glove could also lighten a yellow LED. It is noteworthy that the yellow LED would neither extinguish nor diminish brightness with finger curved from 0° to 135°. These demonstrations indicate that the A-MoO_3-x_/rGO hybrid fibers are promising to be applied in future wearable electronic devices to serve as main/emergency power supply as well as adapt to human daily movements.

## 3. Discussion

In summary, the strengthened Jahn-Teller effect induced by the accumulation of Mo^5+^ was utilized for the phase engineering of *α*-MoO_3_ and A-MoO_3-x_/rGO hybrid fiber electrode was successfully optimized *via* the controllable introduction of oxygen vacancies. In favor of amorphous MoO_3-x_ nanoparticles with appropriate oxygen vacancy content evenly anchored on 3D interconnected graphene networks, the obtained hybrid fiber delivered an ultrahigh *C*_*V*_ of 921.5 F cm^−3^ together with outstanding cycling stability and flexibility, endowing the assembled FSC superior energy storage capability. The universality of the Jahn-Teller effect in crystal engineering other transitional metal oxide needs further investigation, which will be beneficial towards mass manufacturing high-performance hybrid fibers for future wearable energy storage systems.

## 4. Materials and Methods

### 4.1. Synthesis of GO Nanosheets

A modified Hummers' method was employed to synthesize GO nanosheets [60]. Briefly, expandable graphite flakes (1.0 g), NaNO_3_ (0.5 g), and concentrated H_2_SO_4_ (46 mL) were mixed and placed in an ice bath under stirring. KMnO_4_ (5.0 g) was slowly added to keep the reaction temperature below 20°C. Then, the mixture was heated to 35 ± 1°C and maintained for 8.0 h before adding deionized water (46 mL). 4 mL H_2_O_2_ (30 wt%) was subsequently added to the resultant suspension; then, GO nanosheets were collected and washed thoroughly.

### 4.2. Synthesis of *α*-MoO_3_ Belts

Typically, 1.0 g Mo powder was firstly dissolved in 15 mL H_2_O_2_ (20 wt%) to obtain a light yellow solution. The mixture was hydrothermally heated at 220°C for 15.0 h, and the precipitate was collected.

### 4.3. Synthesis of A-MoO_3-x_/rGO Hybrid Fibers

Typically, GO aqueous solution (4 mg mL^−1^) was mixed with *α*-MoO_3_ belts and the mass ratio was controlled 2 : 1. After violently shaking, the mixture became homogeneous and subsequently was injected into a glass capillary with an inner diameter of ~1.0 mm. After being sealed at both ends using a welding torch, the glass capillaries were heated in an electric oven at 160°C for 6.0 h. The obtained hydrogel fibers (self-assembled) were then thoroughly washed using deionized water and dried at 60°C in a vacuum oven.

### 4.4. Material Characterization

Field-emission scanning electron microscope (FESEM; JEOL, JSM-7800F) and transmission electron microscope (TEM; JEOL, 1400 PLUS) were employed for morphology characterizations. X-ray diffraction (XRD; smartlab; Cu K*α* radiation, *λ* = 1.5406 Å) was used for identifying crystal structure. The confocal micro-Raman system (WITEC Alpha 300 M^+^) was performed using a diode laser of 633 nm at ambient conditions. X-ray photoelectron spectroscopy (XPS) was carried out on a PHI Quantera spectrometer. Tensile tester (HY-0350) and Keithley 2400 were used for measuring the failure strength and electrical conductivity of fibers.

### 4.5. DFT Calculations

Vienna Ab initio Simulation Package (VASP) was adopted for theoretical calculations based on a gradient approximation described by Perdew-Burke-Ernzerhof (PBE) [61–63], ab initio molecular dynamics for open-shell transition metals, and ab initio molecular dynamics for open-shell transition metals. The wavefunctions in the core region were described by the projector augmented wave (PAW) method [64]. 10^−5^ eV and 0.02 eV Å^−1^ were, respectively, set for total energy and the Hellmann-Feynman force on each relaxed atom during geometry optimization. In order to exterminate any interaction, a spacing of 10 Å was chosen between two nanosheets. The activation barriers and transition states were determined by the climbing image nudged elastic band (NEB) method [65].

### 4.6. Electrochemical Measurement of Single Electrodes

The electrochemical performance of A-MoO_3-x_/rGO hybrid fiber was tested in a three-electrode configuration with a reference electrode of Ag/AgCl (saturated KCl aqueous solution), a counter electrode of Pt plate, and the electrolyte of 1 M H_2_SO_4_. CV, and GCD measurements were performed on an electrochemical workstation (CHI660D, Chenhua). The fiber volume (*V*_we_) was obtained according to *V*_we_ = *L* × *π* × (*D*/2)^2^, where *L* represents fiber length while *D* is fiber diameter. The electrochemical performance of pristine *α*-MoO_3_ was also measured in the same three-electrode system.

### 4.7. Electrochemical Characterization of FSCs

Two ~1.5 cm long A-MoO_3-x_/rGO hybrid fibers (diameter of ~50 *μ*m) were placed closely on a polymeric (PET) substrate, on top of which H_2_SO_4_/PVA gel was coated. Ag paste was applied on one end of fiber electrode for better electrical connection. The electrochemical characterization and capacitance calculation of FSCs are similar to that of a single electrode.

## Data Availability

The data used to support the findings of this study are included within the article and supplementary information files and/or may be requested from the authors.

## Supplementary Materials

Supplementary Materials Figure S1: morphology characterization of *α*-MoO_3_ belts. Figure S2: crystal interlayer spacing analysis of *α*-MoO_3_ belts. Figure S3: crystal structure characterizations of *α*-MoO_3_ belts and the obtained A-MoO_3-x_/rGO hybrid fiber. Figure S4: crystal structure characterizations of hydrothermal treated *α*-MoO_3_ belts with different conditions. Figure S5: morphology characterizations of A-MoO_3-x_/rGO hybrid fibers obtained at different synthetic time. Figure S6: crystal structure characterizations of A-MoO_3-x_/rGO hybrid fibers obtained at different synthetic time. Figure S7: electrochemical properties of A-MoO_3-x_/rGO hybrid fibers obtained at different synthetic conditions. Figure S8: CV profiles of the pristine *α*-MoO_3_ belts. Figure S9: electrochemical properties of the optimized A-MoO_3-x_/rGO hybrid fiber. Figure S10: Nyquist plots of bare rGO fiber and A-MoO_3-x_/rGO hybrid fibers, respectively. Figure S11: analysis of capacitance contribution of optimized A-MoO_3-x_/rGO hybrid fiber. Figure S12: schematic illustration of the ion transport channels within *α*-MoO_3_ crystals and A-MoO_3-x_, respectively.
