# Supplementary material for: The Jahn-Teller Effect for Amorphization of Molybdenum Trioxide towards High-Performance Fiber Supercapacitor
Source: Research (Wash D C). 2021 Mar 29;2021:6742715. doi: 10.34133/2021/6742715 (PMC8025085; doi:10.34133/2021/6742715)
Supplement: Supplementary Materials — Figure S1: morphology characterization of α-MoO3 belts. Figure S2: crystal interlayer spacing analysis of α-MoO3 belts. Figure S3: crystal structure characterizations of α-MoO3 belts and the obtained A-MoO3-x/rGO hybrid fiber. Figure S4: crystal structure characterizations of hydrothermal treated α-MoO3 belts with different conditions. Figure S5: morphology characterizations of A-MoO3-x/rGO hybrid fibers obtained at different synthetic time. Figure S6: crystal structure characterizations of A-MoO3-x/rGO hybrid fibers obtained at different synthetic time. Figure S7: electrochemical properties of A-MoO3-x/rGO hybrid fibers obtained at different synthetic conditions. Figure S8: CV profiles of the pristine α-MoO3 belts. Figure S9: electrochemical properties of the optimized A-MoO3-x/rGO hybrid fiber. Figure S10: Nyquist plots of bare rGO fiber and A-MoO3-x/rGO hybrid fibers, respectively. Figure S11: analysis of capacitance contribution of optimized A-MoO3-x/rGO hybrid fiber. Figure S12: schematic illustration of the ion transport channels within α-MoO3 crystals and A-MoO3-x, respectively. [file 6742715.f1.zip › Supplementary Materials-Research-20210318.docx]

Supplementary Materials for

# The Jahn-Teller Effect for Amorphization of Molybdenum Trioxide towards High Performance Fiber Supercapacitor

Chenyang Yu,^†^ Hai Xu,^†^ Yujiao Gong, Ruyi Chen, Zengyu Hui, Xi Zhao, Yue Sun, Qiang Chen, Jinyuan Zhou, Wenxin Ji, Gengzhi Sun,* and Wei Huang*

**This PDF file includes:**

**Fig. S1.** Morphology characterization of α-MoO_3_ belts.

**Fig. S2.** Crystal interlayer spacing analysis of α-MoO_3_ belts.

**Fig. S3.** Crystal structure characterizations of α-MoO_3_ belts and the obtained A-MoO_3-x_/rGO hybrid fiber.

**Fig. S4.** Crystal structure characterizations of hydrothermal treated α-MoO_3_ belts with different conditions.

**Fig. S5.** Morphology characterizations of A-MoO_3-x_/rGO hybrid fibers obtained at different synthetic time.

**Fig. S6.** Crystal structure characterizations of A-MoO_3-x_/rGO hybrid fibers obtained at different synthetic time.

**Fig. S7.** Electrochemical properties of A-MoO_3-x_/rGO hybrid fibers obtained at different synthetic conditions.

**Fig. S8.** CV profiles of the pristine α-MoO_3_ belts.

**Fig. S9.** Electrochemical properties of the optimized A-MoO_3-x_/rGO hybrid fiber.

**Fig. S10.** Nyquist plots of bare rGO fiber and A-MoO_3-x_/rGO hybrid fibers, respectively.

**Fig. S11.** Analysis of capacitance contribution of optimized A-MoO_3-x_/rGO hybrid fiber.

**Fig. S12.** Schematic illustration of the ions transport channels within α-MoO_3_ crystals and A-MoO_3-x_, respectively.


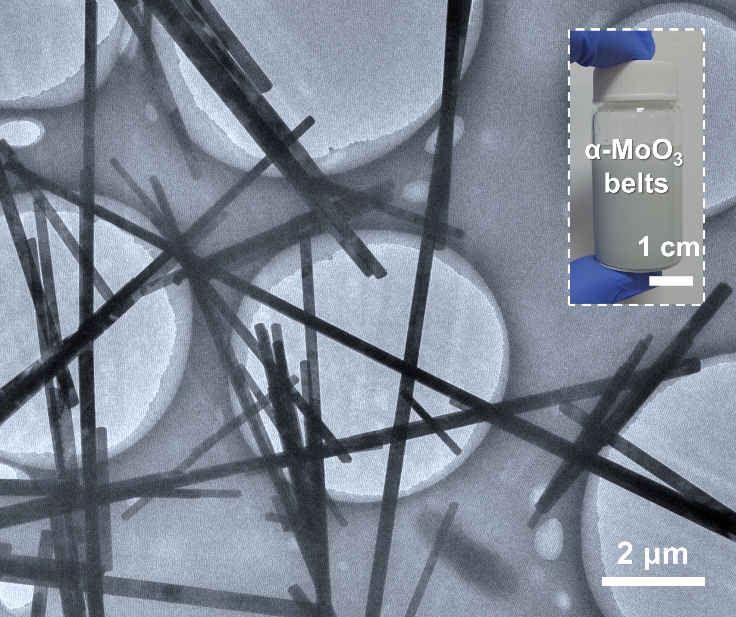


**Fig. S1. Morphology characterization of α-MoO_3_ belts.** TEM image of α-MoO_3_ belts. The inset is the photo image of the α-MoO_3_ belts suspension.


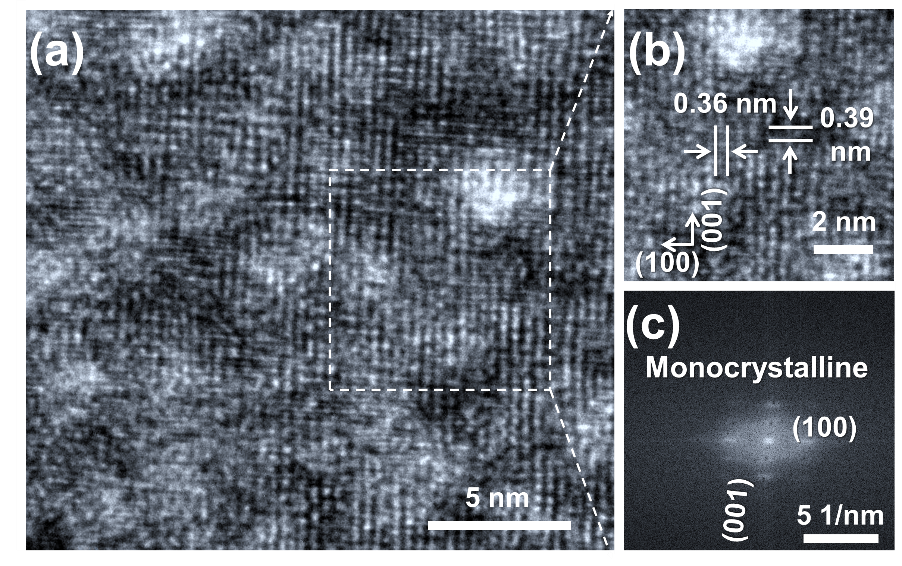


**Fig. S2.** **Crystal interlayer spacing analysis of α-MoO_3_ belts.** (a, b) HRTEM and (c) the corresponding FFT images of α-MoO_3_ belts, respectively.


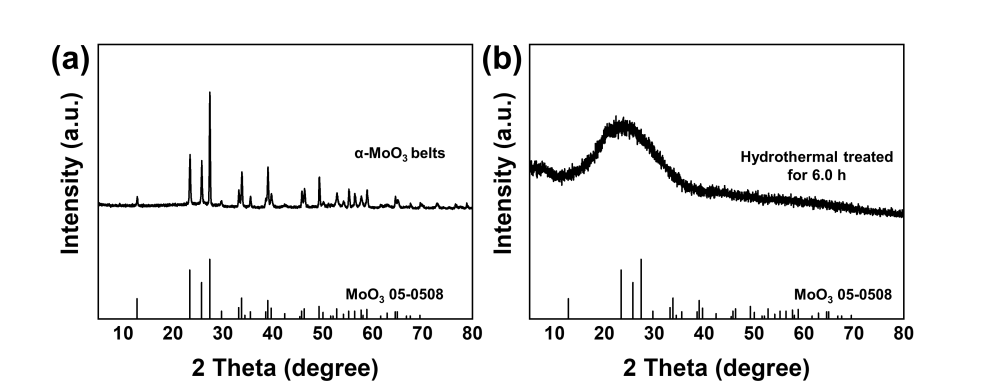


**Fig. S3.** **Crystal structure characterizations of α-MoO_3_ belts and the obtained A-MoO_3-x_/rGO hybrid fiber.** XRD patterns of (a) α-MoO_3_ belts and (b) the A-MoO_3-x_/rGO hybrid fiber with 6.0 h hydrothermal treated.


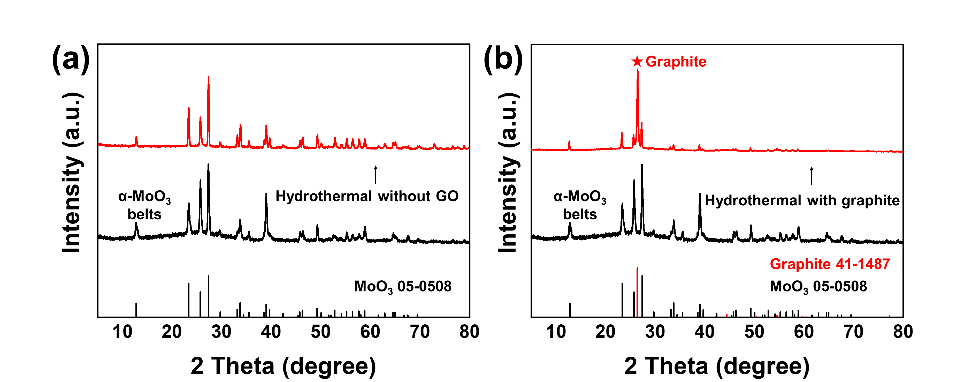


**Fig. S4.** **Crystal structure characterizations of hydrothermal treated α-MoO_3_ belts with different conditions.** XRD patterns of hydrothermal treated α-MoO_3_ belts (a) without GO or any other additives and (b) with graphite powder.


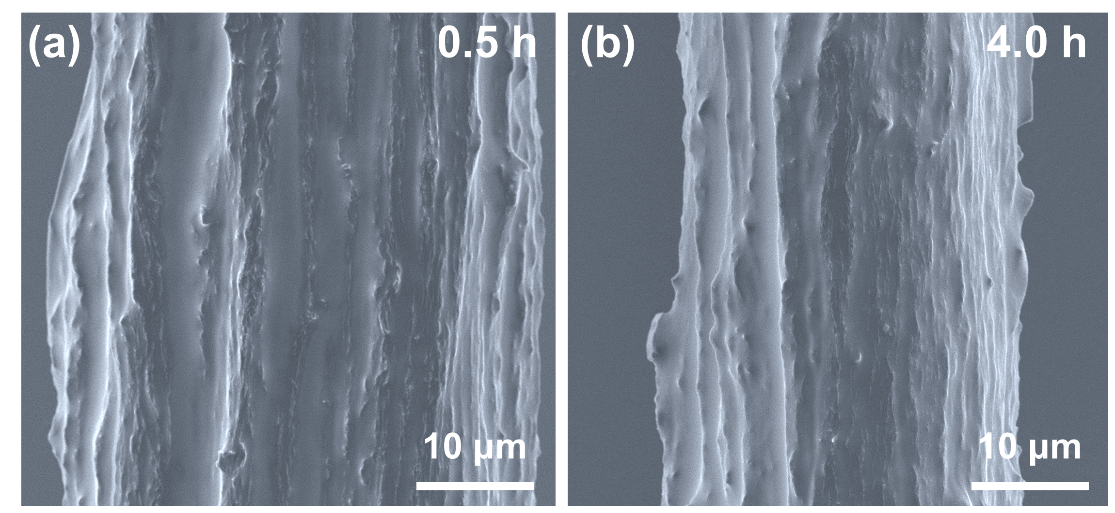


**Fig. S5.** **Morphology characterizations of A-MoO_3-x_/rGO hybrid fibers obtained at different synthetic time.** SEM images of A-MoO_3-x_/rGO hybrid fiber obtained at (a) 0.5 h and (b) 4.0 h, respectively.


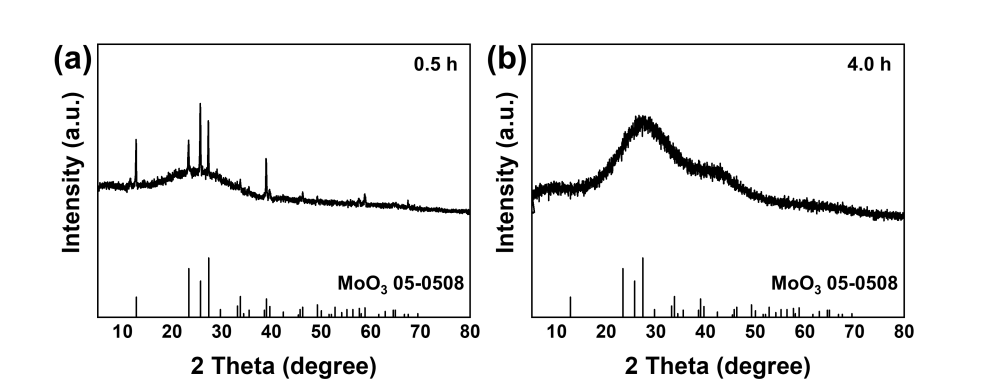


**Fig. S6.** **Crystal structure characterizations of A-MoO_3-x_/rGO hybrid fibers obtained at different synthetic time.** XRD patterns of A-MoO_3-x_/rGO hybrid fibers at different synthetic time of (a) 0.5 h and (b) 4.0 h, respectively.


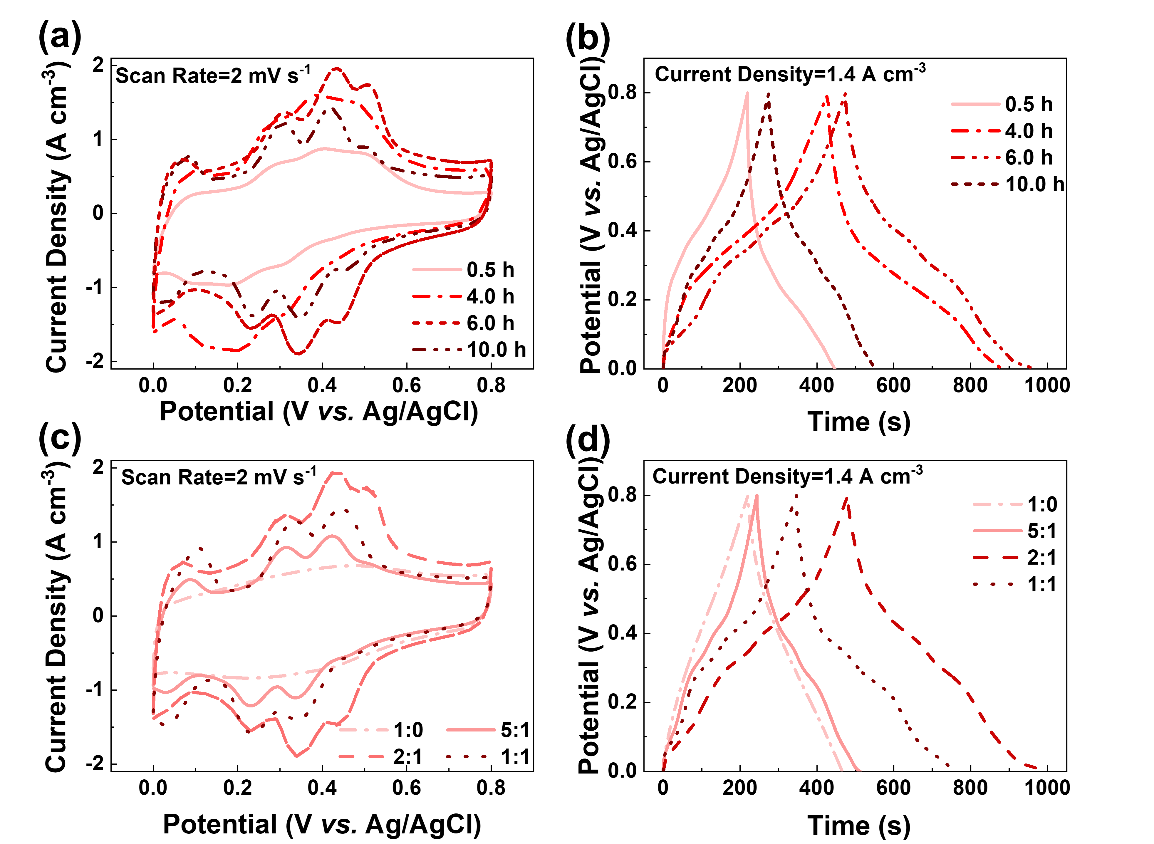


**Fig. S7.** **Electrochemical properties of A-MoO_3-x_/rGO hybrid fibers obtained at different synthetic conditions.** (a) CV curves and (b) GCD profiles of A-MoO_3-x_/rGO hybrid fibers at different synthetic time, the scan rate and current density were 2 mV s^-1^ and 1.4 A cm^-3^, respectively. (c) CV curves and (d) GCD profiles of A-MoO_3-x_/rGO hybrid fibers at different feeding ratio of GO and α-MoO_3_ belts, the scan rate and current density were 2 mV s^-1^ and 1.4 A cm^-3^, respectively.


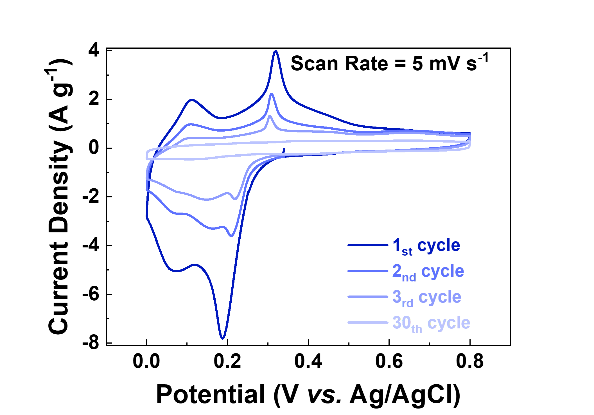


**Fig. S8.** **CV profiles of the pristine α-MoO_3_ belts.**


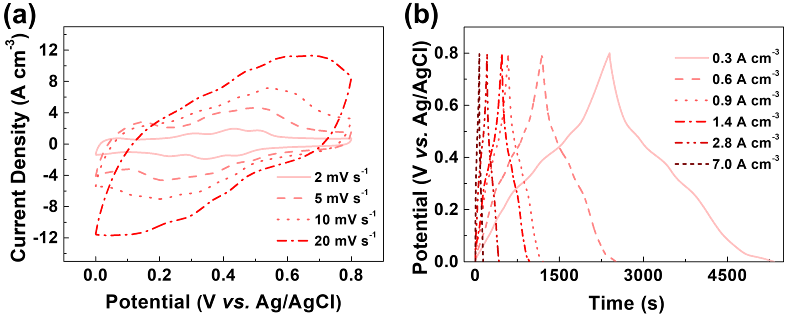


**Fig. S9.** **Electrochemical properties of the optimized A-MoO_3-x_/rGO hybrid fiber.** (a) CV and (b) GCD profiles of optimized A-MoO_3-x_/rGO hybrid fiber at different scan rates and charge-discharge current densities, respectively.


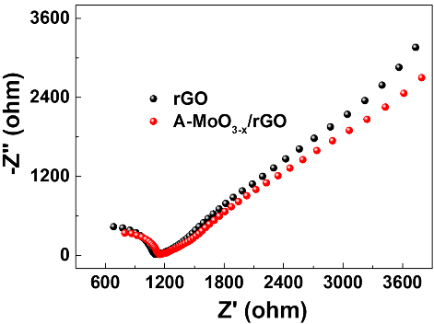


**Fig. S10.** **Nyquist plots of bare rGO fiber and A-MoO_3-x_/rGO hybrid fibers, respectively.**


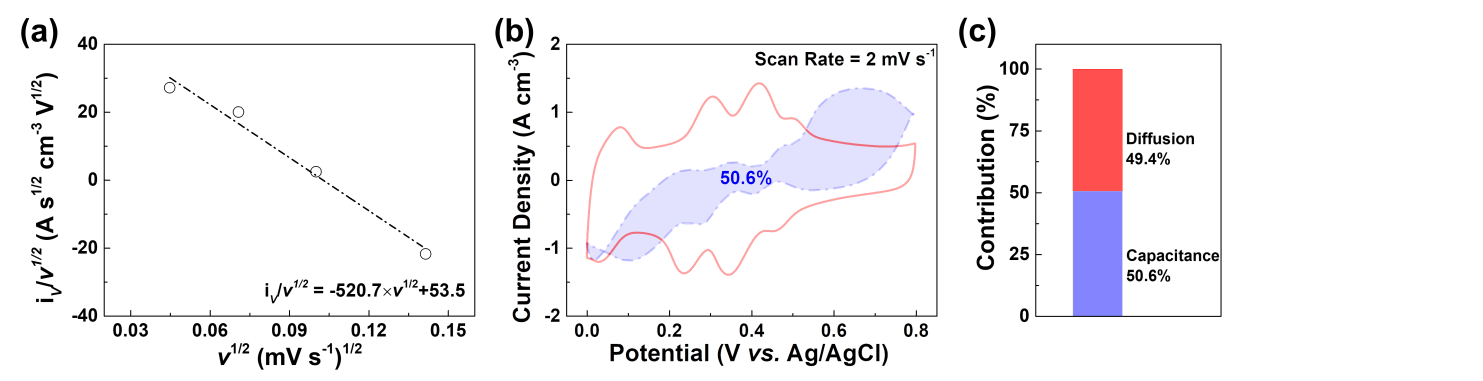


**Fig. S11.** **Analysis of capacitance contribution of optimized A-MoO_3-x_/rGO hybrid fiber.** (a) Plot of current density (*i_V_*) divided by square root of scan rate (*v*^1/2^) against *v*^1/2^ of optimized A-MoO_3-x_/rGO hybrid fiber using the cathodic current density at the potential of 0.3 V. (b, c) Analysis of capacitance contribution of the optimized A-MoO_3-x_/rGO hybrid fiber.


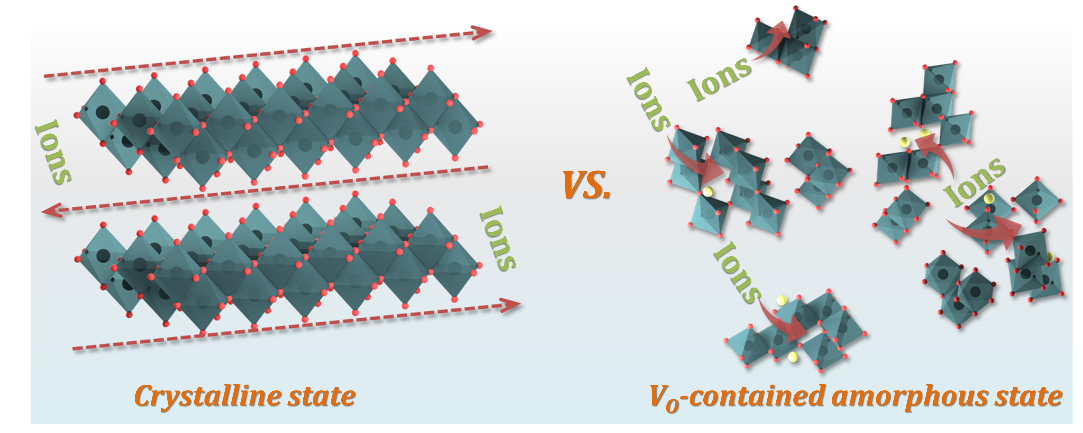


**Fig. S12.** **Schematic illustration of the ions transport channels within α-MoO_3_ crystals and A-MoO_3-x_, respectively.**
