# Supplementary figures and images for: The Jahn-Teller Effect for Amorphization of Molybdenum Trioxide towards High-Performance Fiber Supercapacitor
Source: Research (Wash D C). 2021 Mar 29;2021:6742715. doi: 10.34133/2021/6742715 (PMC8025085; doi:10.34133/2021/6742715)

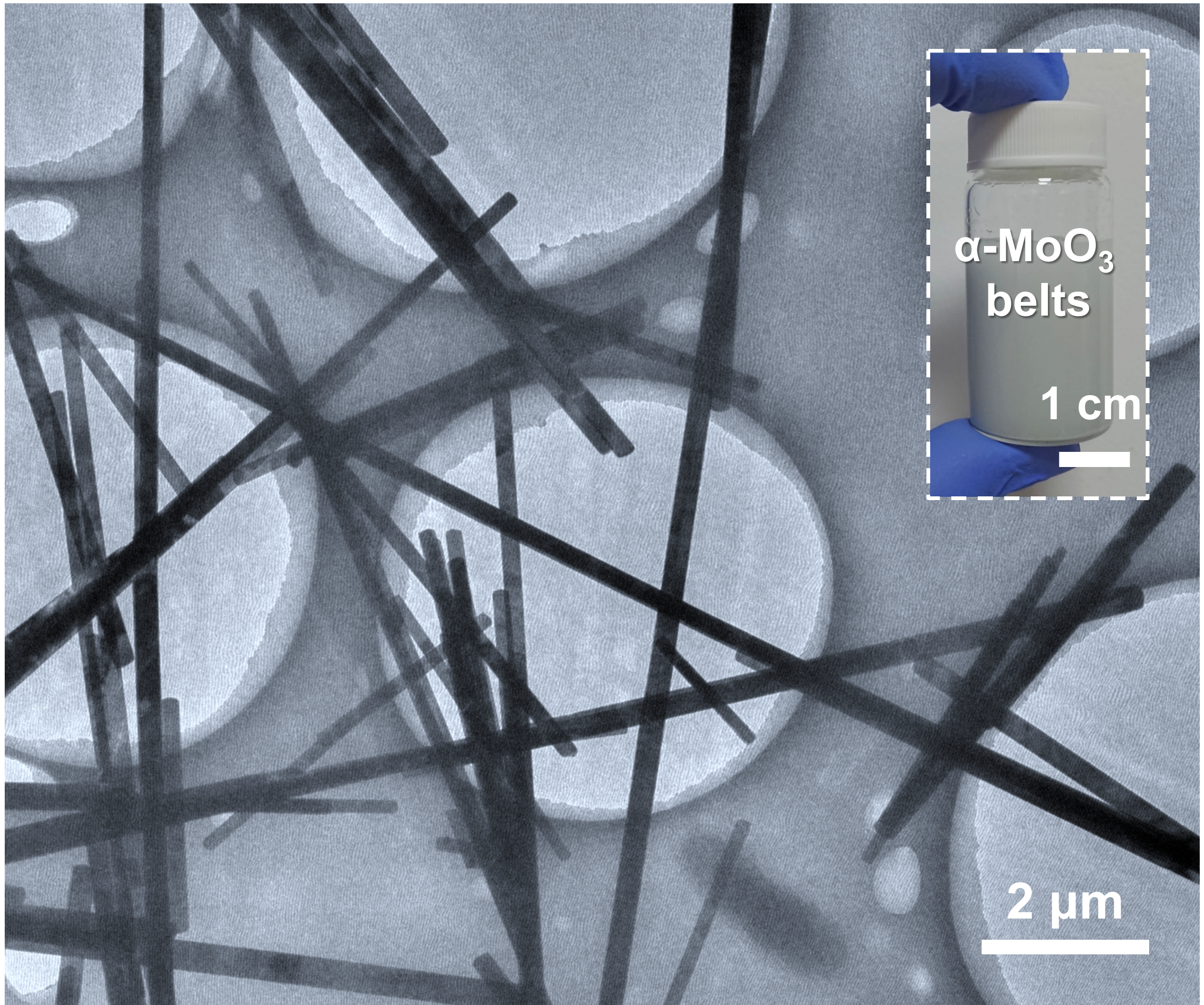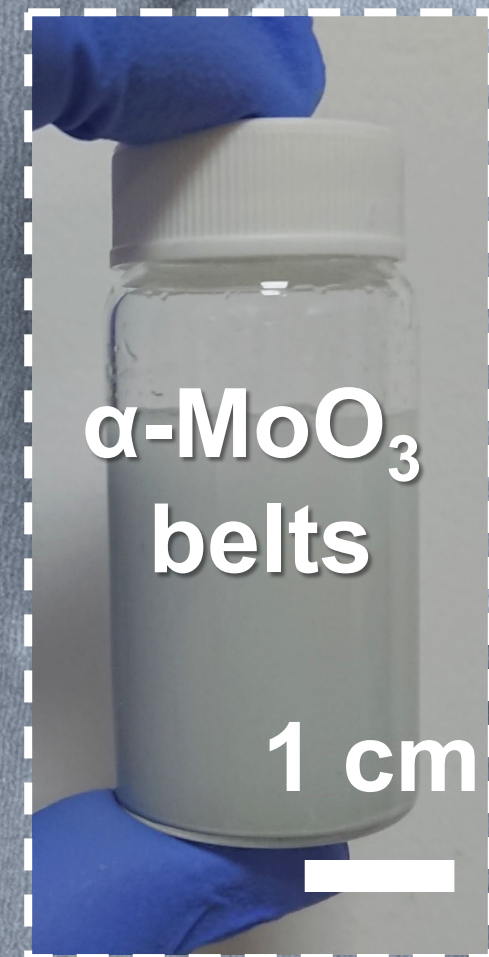

Supplement: Supplementary Materials — Figure S1: morphology characterization of α-MoO3 belts. Figure S2: crystal interlayer spacing analysis of α-MoO3 belts. Figure S3: crystal structure characterizations of α-MoO3 belts and the obtained A-MoO3-x/rGO hybrid fiber. Figure S4: crystal structure characterizations of hydrothermal treated α-MoO3 belts with different conditions. Figure S5: morphology characterizations of A-MoO3-x/rGO hybrid fibers obtained at different synthetic time. Figure S6: crystal structure characterizations of A-MoO3-x/rGO hybrid fibers obtained at different synthetic time. Figure S7: electrochemical properties of A-MoO3-x/rGO hybrid fibers obtained at different synthetic conditions. Figure S8: CV profiles of the pristine α-MoO3 belts. Figure S9: electrochemical properties of the optimized A-MoO3-x/rGO hybrid fiber. Figure S10: Nyquist plots of bare rGO fiber and A-MoO3-x/rGO hybrid fibers, respectively. Figure S11: analysis of capacitance contribution of optimized A-MoO3-x/rGO hybrid fiber. Figure S12: schematic illustration of the ion transport channels within α-MoO3 crystals and A-MoO3-x, respectively. [file 6742715.f1.zip › Yu_Figure of SI_Figure S1.pdf]

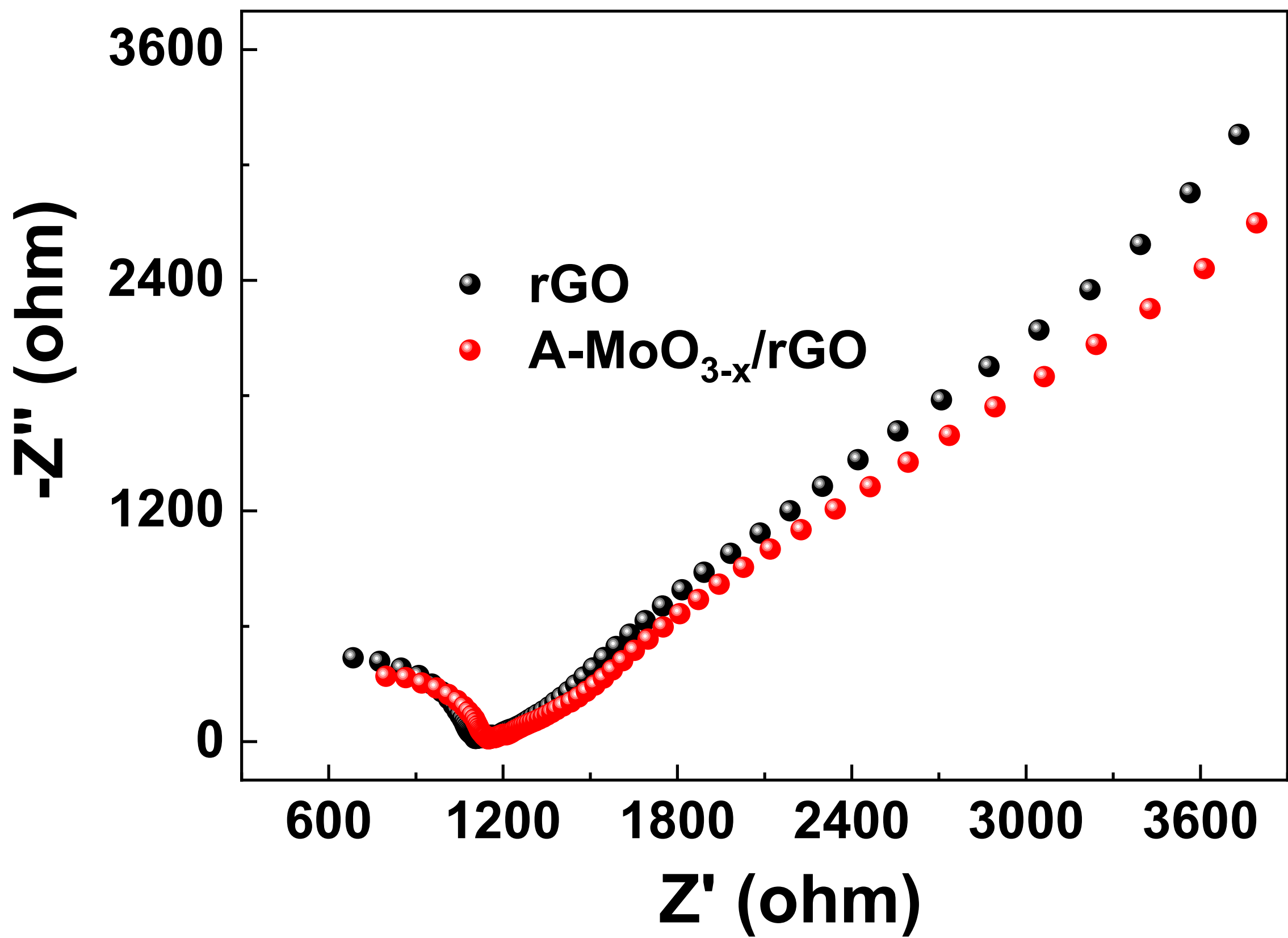

Supplement: Supplementary Materials — Figure S1: morphology characterization of α-MoO3 belts. Figure S2: crystal interlayer spacing analysis of α-MoO3 belts. Figure S3: crystal structure characterizations of α-MoO3 belts and the obtained A-MoO3-x/rGO hybrid fiber. Figure S4: crystal structure characterizations of hydrothermal treated α-MoO3 belts with different conditions. Figure S5: morphology characterizations of A-MoO3-x/rGO hybrid fibers obtained at different synthetic time. Figure S6: crystal structure characterizations of A-MoO3-x/rGO hybrid fibers obtained at different synthetic time. Figure S7: electrochemical properties of A-MoO3-x/rGO hybrid fibers obtained at different synthetic conditions. Figure S8: CV profiles of the pristine α-MoO3 belts. Figure S9: electrochemical properties of the optimized A-MoO3-x/rGO hybrid fiber. Figure S10: Nyquist plots of bare rGO fiber and A-MoO3-x/rGO hybrid fibers, respectively. Figure S11: analysis of capacitance contribution of optimized A-MoO3-x/rGO hybrid fiber. Figure S12: schematic illustration of the ion transport channels within α-MoO3 crystals and A-MoO3-x, respectively. [file 6742715.f1.zip › Yu_Figure of SI_Figure S10.pdf]

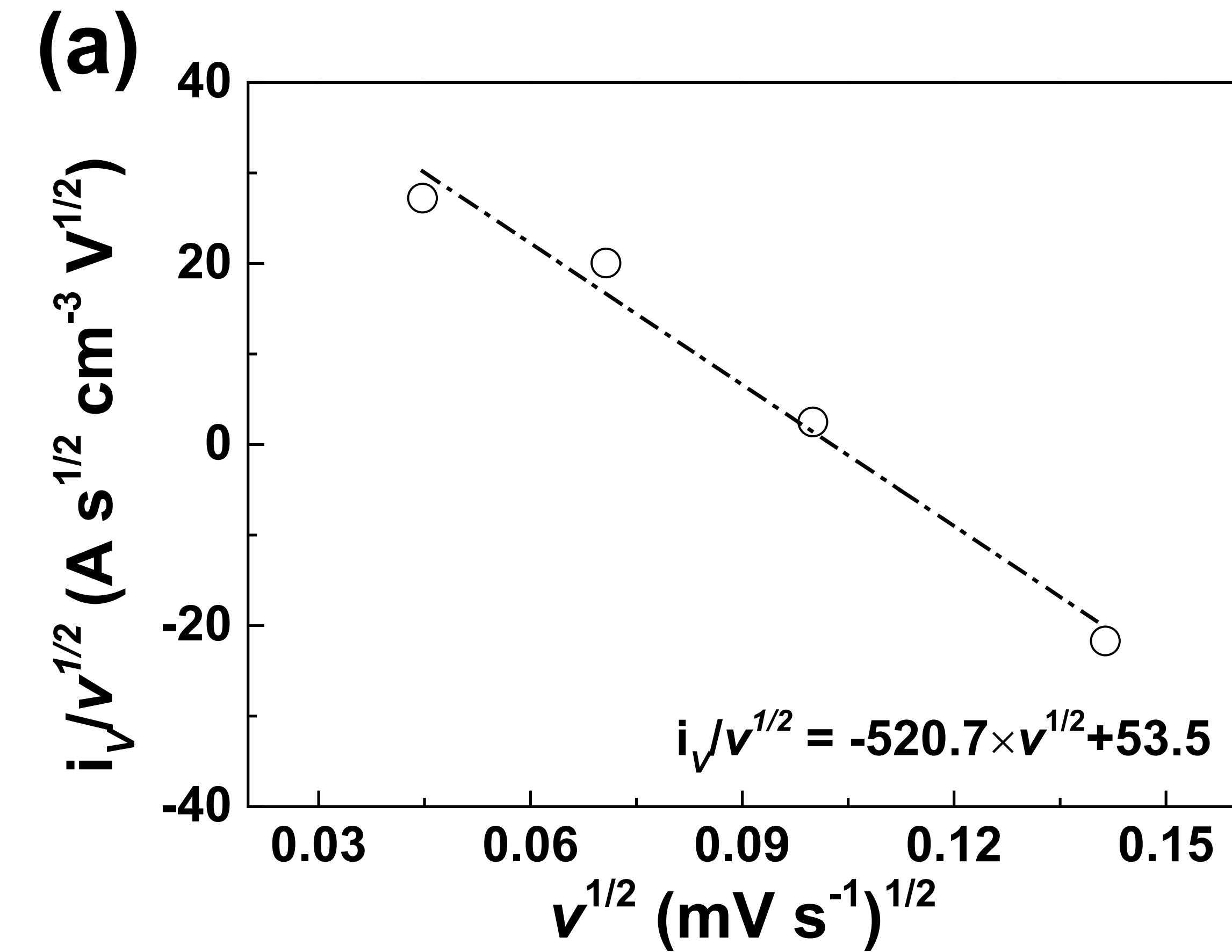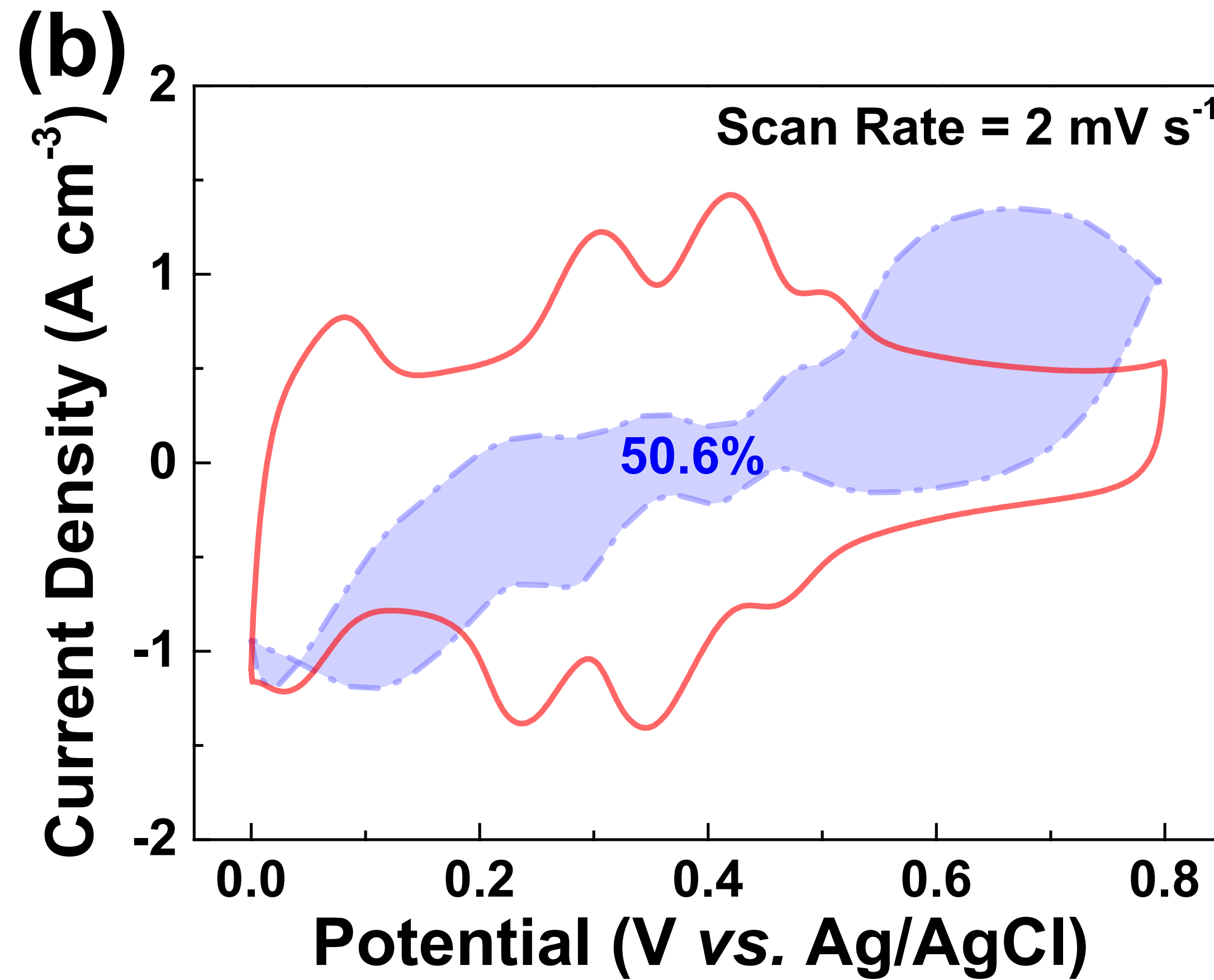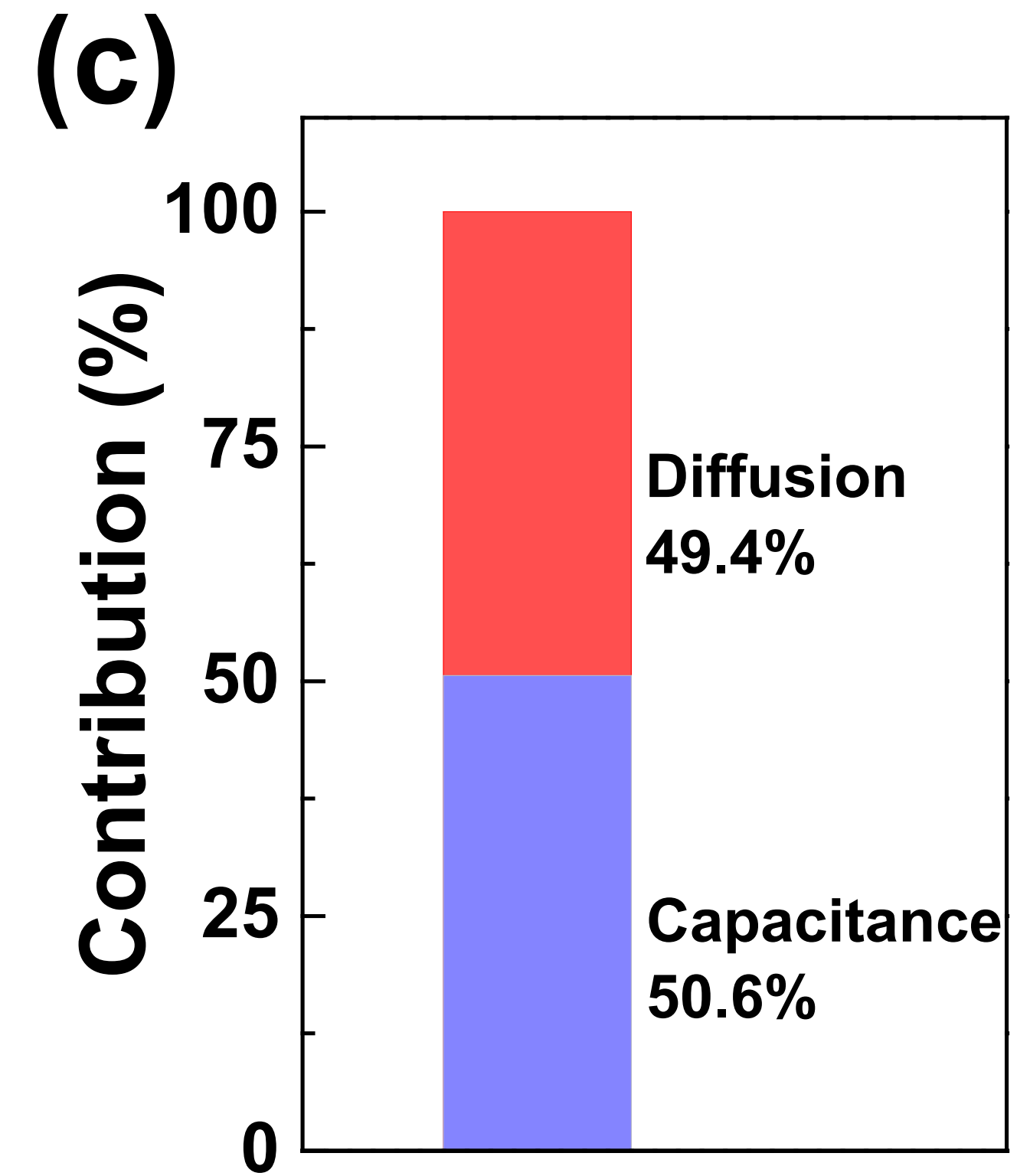

Supplement: Supplementary Materials — Figure S1: morphology characterization of α-MoO3 belts. Figure S2: crystal interlayer spacing analysis of α-MoO3 belts. Figure S3: crystal structure characterizations of α-MoO3 belts and the obtained A-MoO3-x/rGO hybrid fiber. Figure S4: crystal structure characterizations of hydrothermal treated α-MoO3 belts with different conditions. Figure S5: morphology characterizations of A-MoO3-x/rGO hybrid fibers obtained at different synthetic time. Figure S6: crystal structure characterizations of A-MoO3-x/rGO hybrid fibers obtained at different synthetic time. Figure S7: electrochemical properties of A-MoO3-x/rGO hybrid fibers obtained at different synthetic conditions. Figure S8: CV profiles of the pristine α-MoO3 belts. Figure S9: electrochemical properties of the optimized A-MoO3-x/rGO hybrid fiber. Figure S10: Nyquist plots of bare rGO fiber and A-MoO3-x/rGO hybrid fibers, respectively. Figure S11: analysis of capacitance contribution of optimized A-MoO3-x/rGO hybrid fiber. Figure S12: schematic illustration of the ion transport channels within α-MoO3 crystals and A-MoO3-x, respectively. [file 6742715.f1.zip › Yu_Figure of SI_Figure S11.pdf]

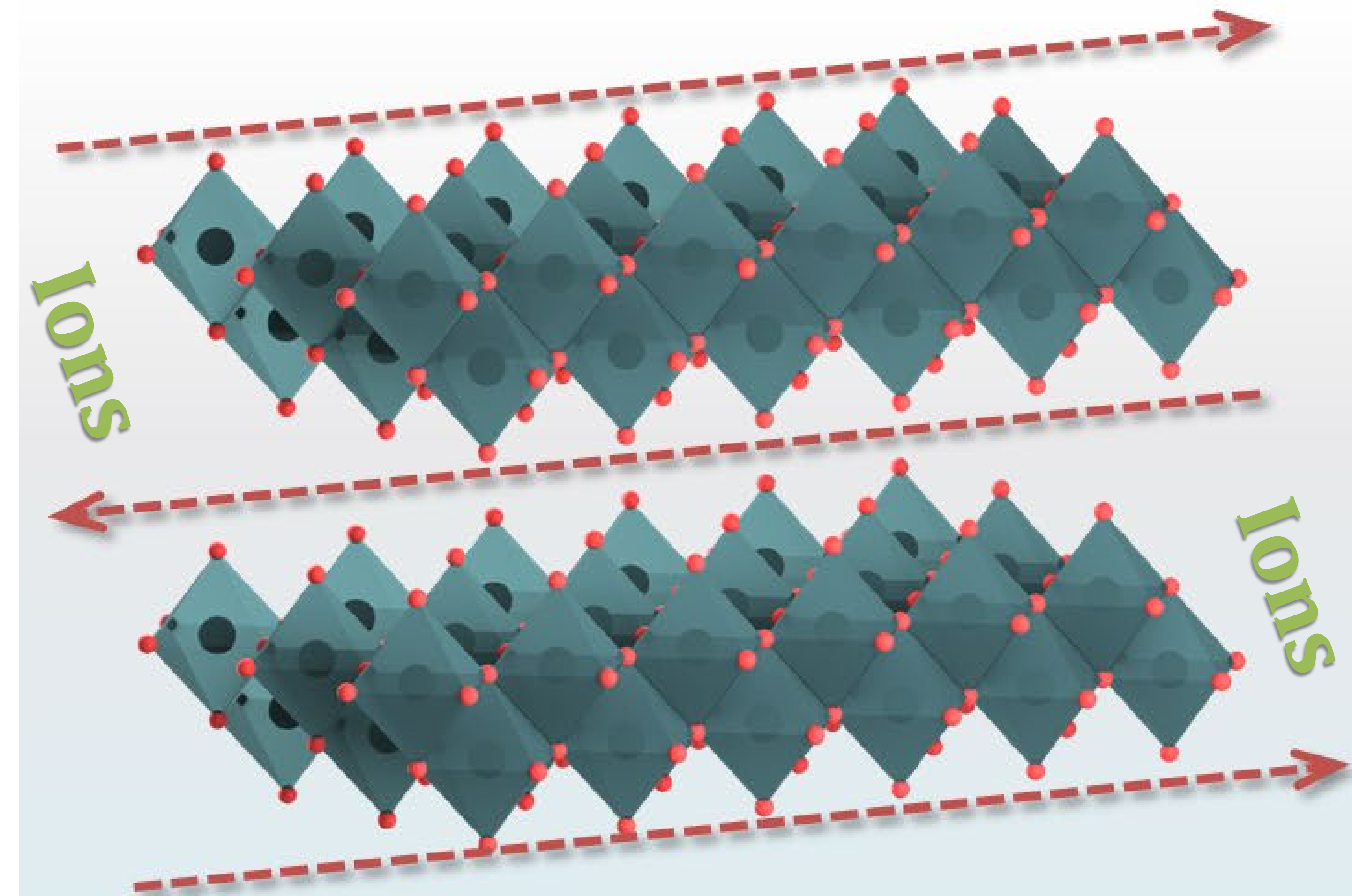

*Crystalline state*

*VS.*

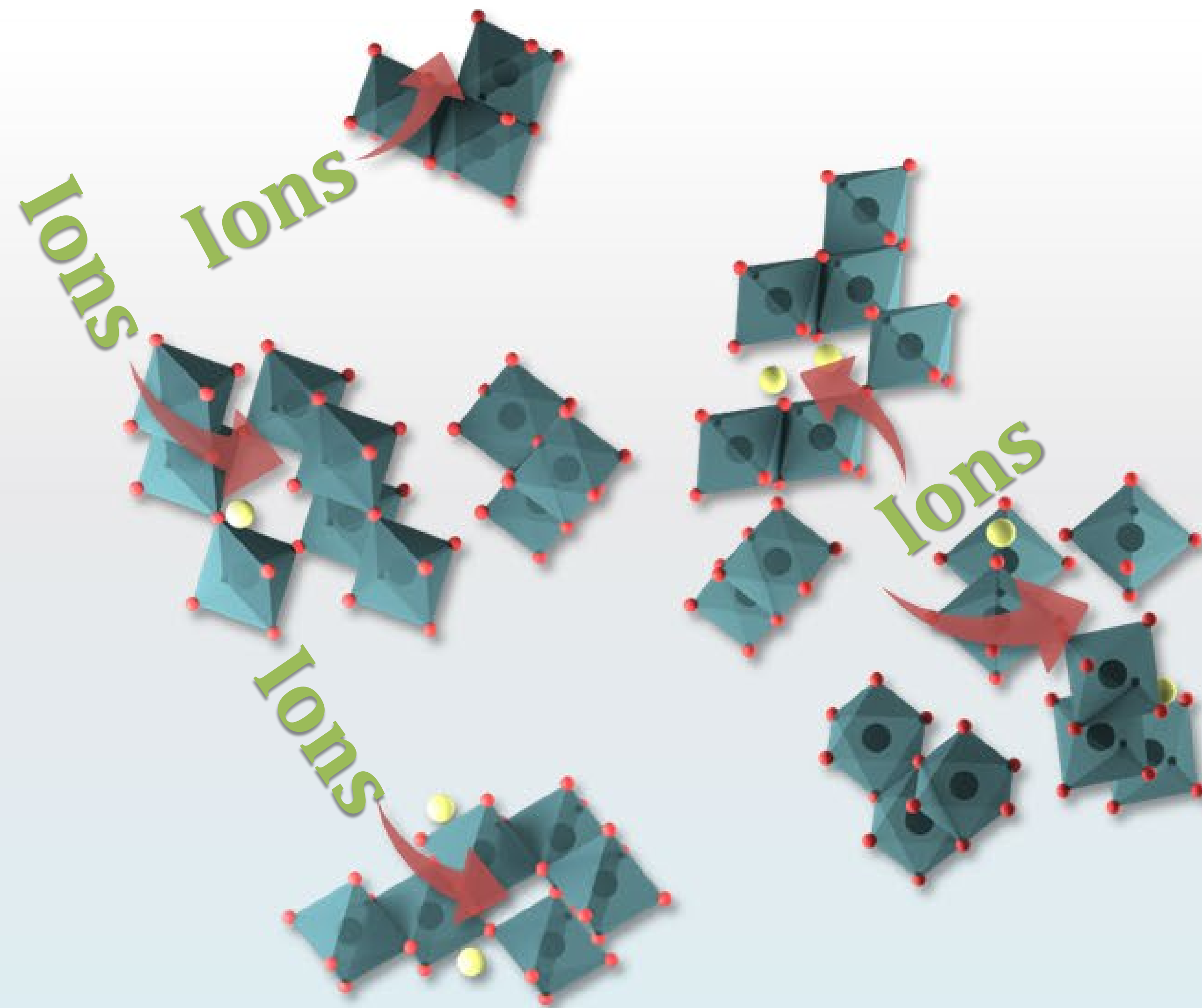

*$V_O$ -contained amorphous state*

Supplement: Supplementary Materials — Figure S1: morphology characterization of α-MoO3 belts. Figure S2: crystal interlayer spacing analysis of α-MoO3 belts. Figure S3: crystal structure characterizations of α-MoO3 belts and the obtained A-MoO3-x/rGO hybrid fiber. Figure S4: crystal structure characterizations of hydrothermal treated α-MoO3 belts with different conditions. Figure S5: morphology characterizations of A-MoO3-x/rGO hybrid fibers obtained at different synthetic time. Figure S6: crystal structure characterizations of A-MoO3-x/rGO hybrid fibers obtained at different synthetic time. Figure S7: electrochemical properties of A-MoO3-x/rGO hybrid fibers obtained at different synthetic conditions. Figure S8: CV profiles of the pristine α-MoO3 belts. Figure S9: electrochemical properties of the optimized A-MoO3-x/rGO hybrid fiber. Figure S10: Nyquist plots of bare rGO fiber and A-MoO3-x/rGO hybrid fibers, respectively. Figure S11: analysis of capacitance contribution of optimized A-MoO3-x/rGO hybrid fiber. Figure S12: schematic illustration of the ion transport channels within α-MoO3 crystals and A-MoO3-x, respectively. [file 6742715.f1.zip › Yu_Figure of SI_Figure S12.pdf]

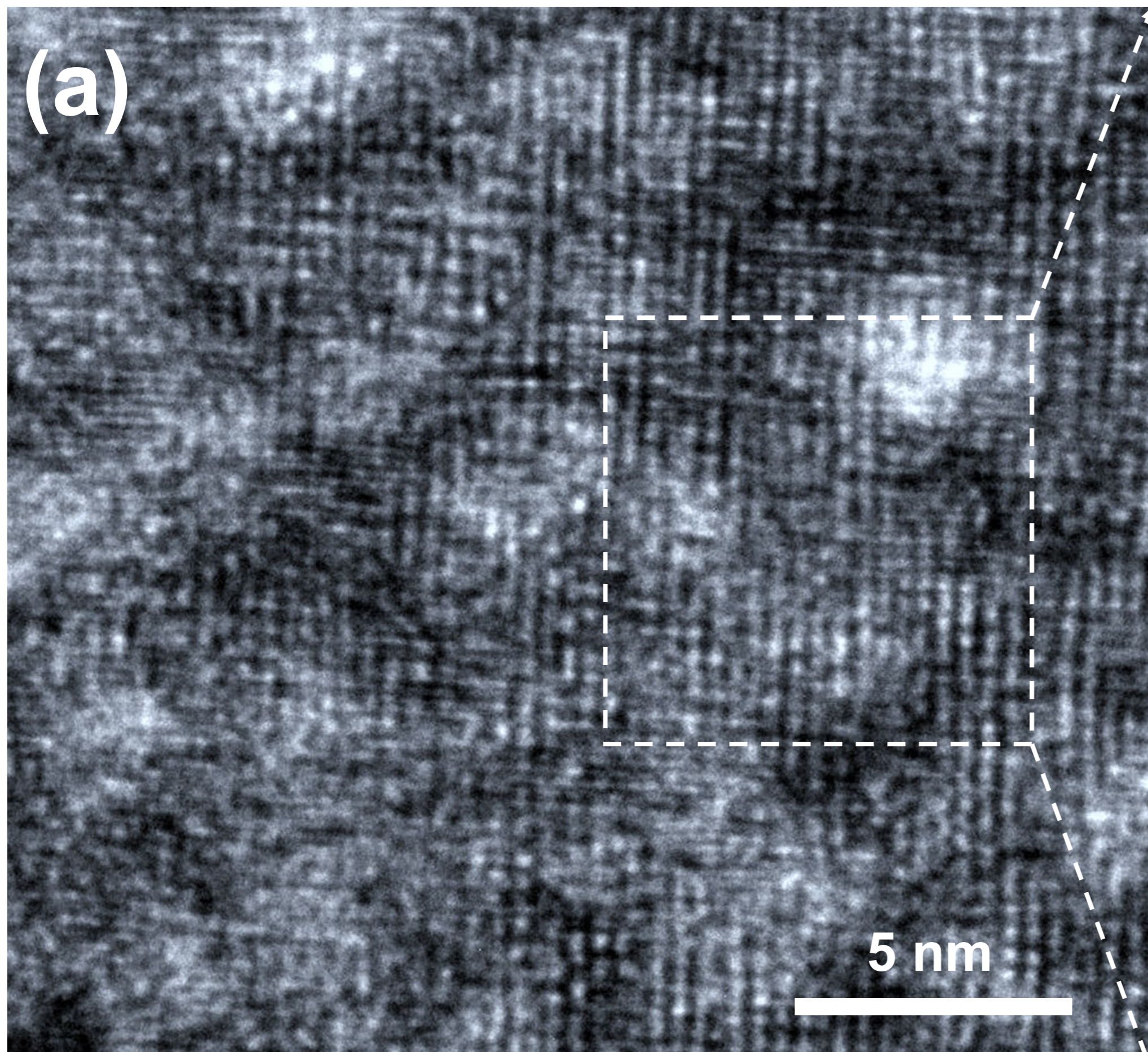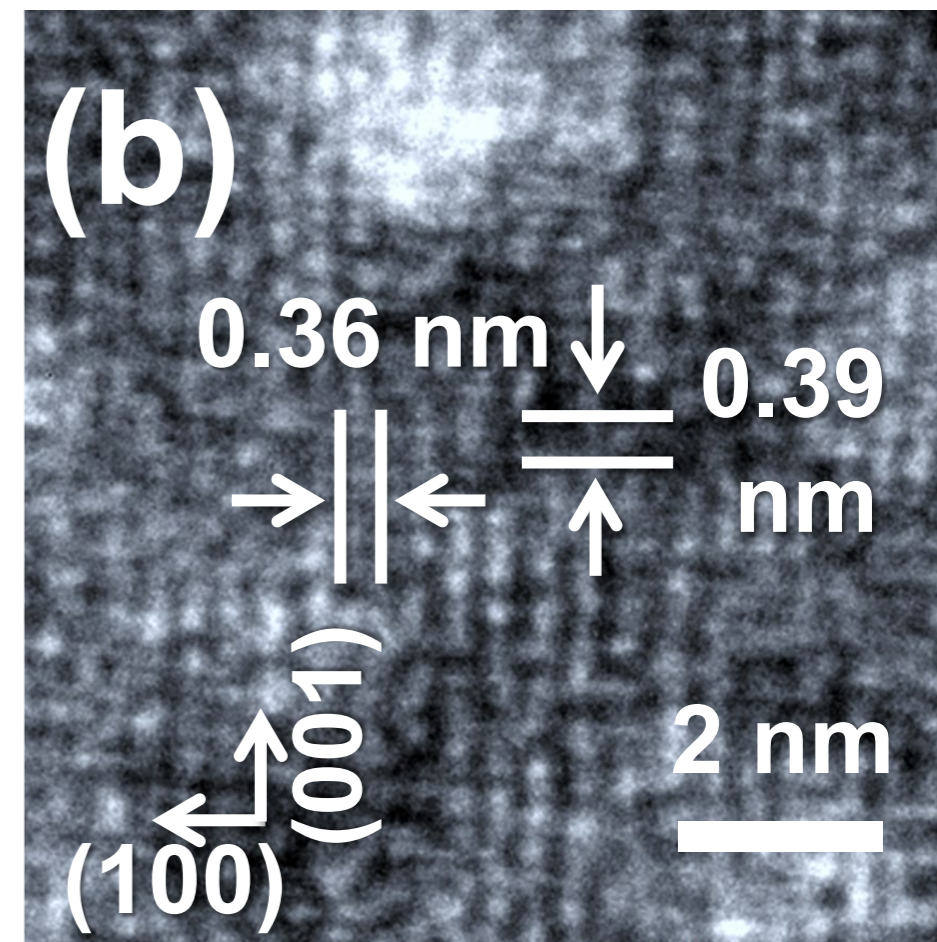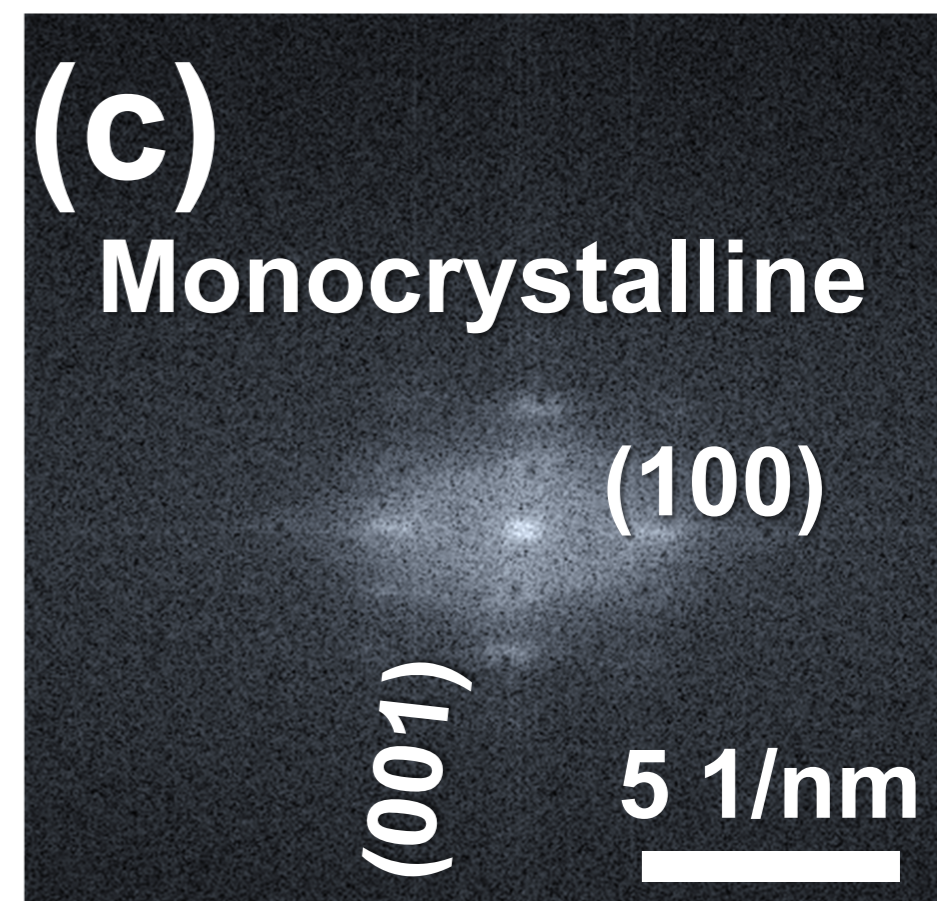

Supplement: Supplementary Materials — Figure S1: morphology characterization of α-MoO3 belts. Figure S2: crystal interlayer spacing analysis of α-MoO3 belts. Figure S3: crystal structure characterizations of α-MoO3 belts and the obtained A-MoO3-x/rGO hybrid fiber. Figure S4: crystal structure characterizations of hydrothermal treated α-MoO3 belts with different conditions. Figure S5: morphology characterizations of A-MoO3-x/rGO hybrid fibers obtained at different synthetic time. Figure S6: crystal structure characterizations of A-MoO3-x/rGO hybrid fibers obtained at different synthetic time. Figure S7: electrochemical properties of A-MoO3-x/rGO hybrid fibers obtained at different synthetic conditions. Figure S8: CV profiles of the pristine α-MoO3 belts. Figure S9: electrochemical properties of the optimized A-MoO3-x/rGO hybrid fiber. Figure S10: Nyquist plots of bare rGO fiber and A-MoO3-x/rGO hybrid fibers, respectively. Figure S11: analysis of capacitance contribution of optimized A-MoO3-x/rGO hybrid fiber. Figure S12: schematic illustration of the ion transport channels within α-MoO3 crystals and A-MoO3-x, respectively. [file 6742715.f1.zip › Yu_Figure of SI_Figure S2.pdf]

**(a)**

Intensity (a.u.)

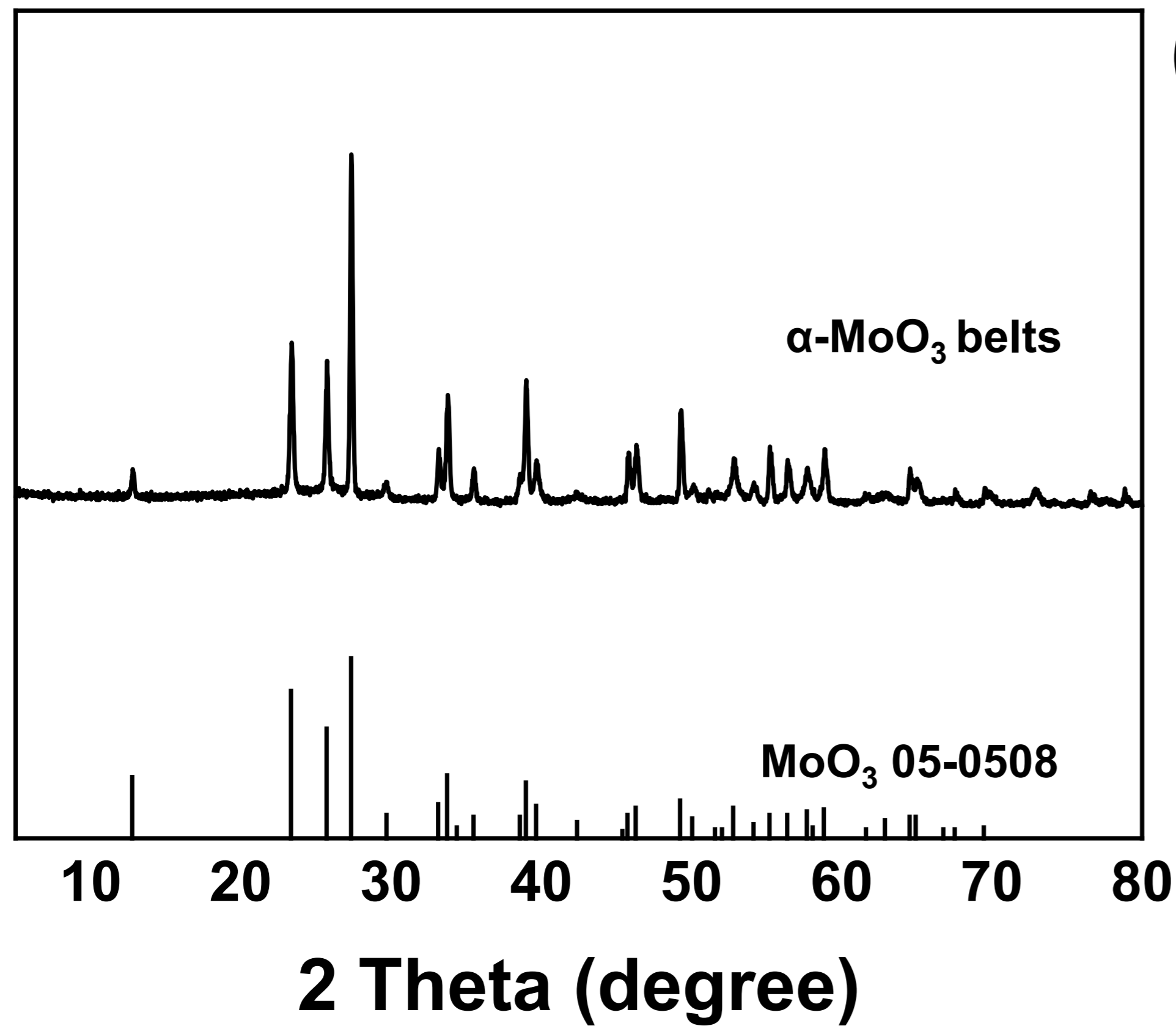

**(b)**

Intensity (a.u.)

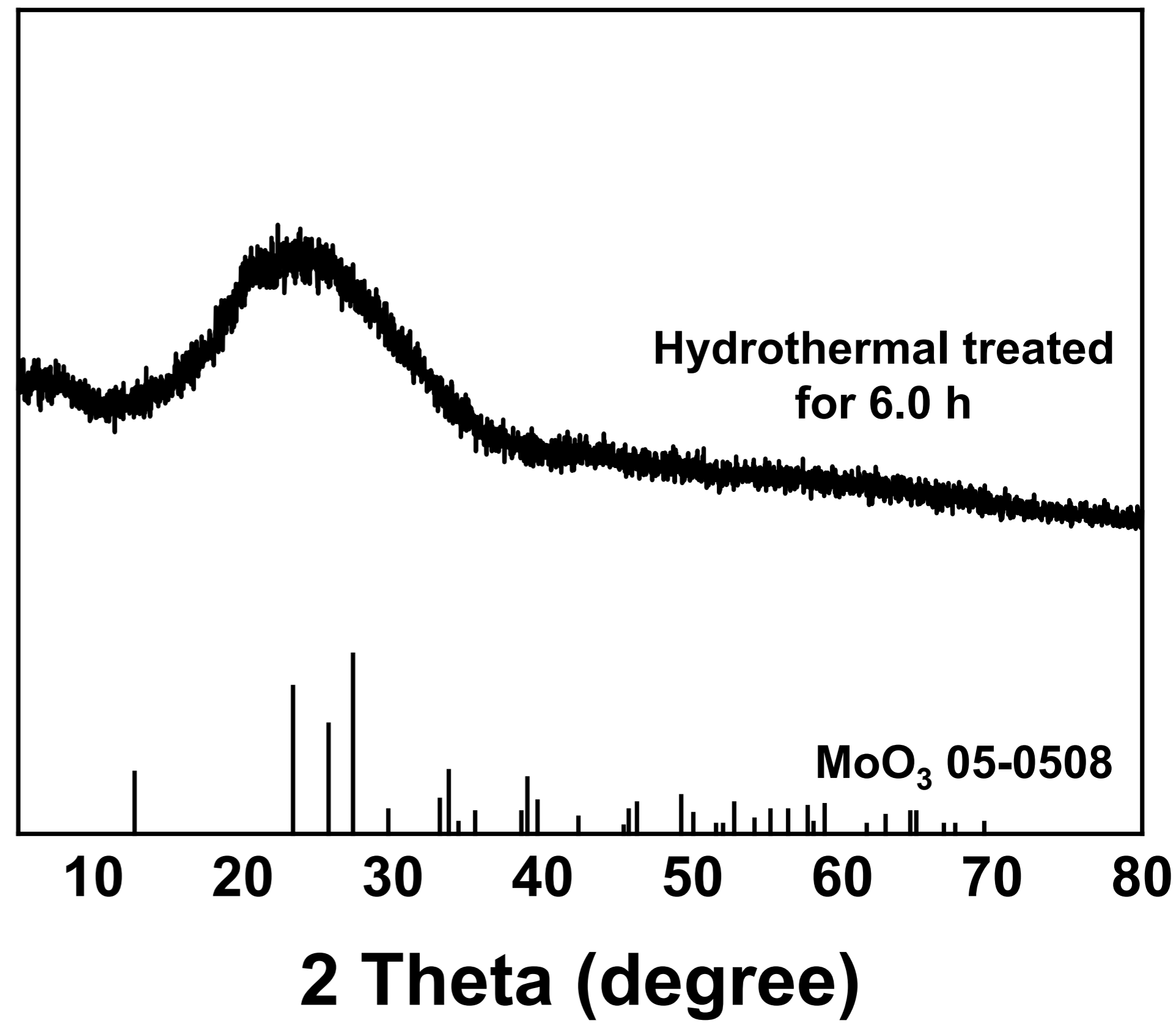

Supplement: Supplementary Materials — Figure S1: morphology characterization of α-MoO3 belts. Figure S2: crystal interlayer spacing analysis of α-MoO3 belts. Figure S3: crystal structure characterizations of α-MoO3 belts and the obtained A-MoO3-x/rGO hybrid fiber. Figure S4: crystal structure characterizations of hydrothermal treated α-MoO3 belts with different conditions. Figure S5: morphology characterizations of A-MoO3-x/rGO hybrid fibers obtained at different synthetic time. Figure S6: crystal structure characterizations of A-MoO3-x/rGO hybrid fibers obtained at different synthetic time. Figure S7: electrochemical properties of A-MoO3-x/rGO hybrid fibers obtained at different synthetic conditions. Figure S8: CV profiles of the pristine α-MoO3 belts. Figure S9: electrochemical properties of the optimized A-MoO3-x/rGO hybrid fiber. Figure S10: Nyquist plots of bare rGO fiber and A-MoO3-x/rGO hybrid fibers, respectively. Figure S11: analysis of capacitance contribution of optimized A-MoO3-x/rGO hybrid fiber. Figure S12: schematic illustration of the ion transport channels within α-MoO3 crystals and A-MoO3-x, respectively. [file 6742715.f1.zip › Yu_Figure of SI_Figure S3.pdf]

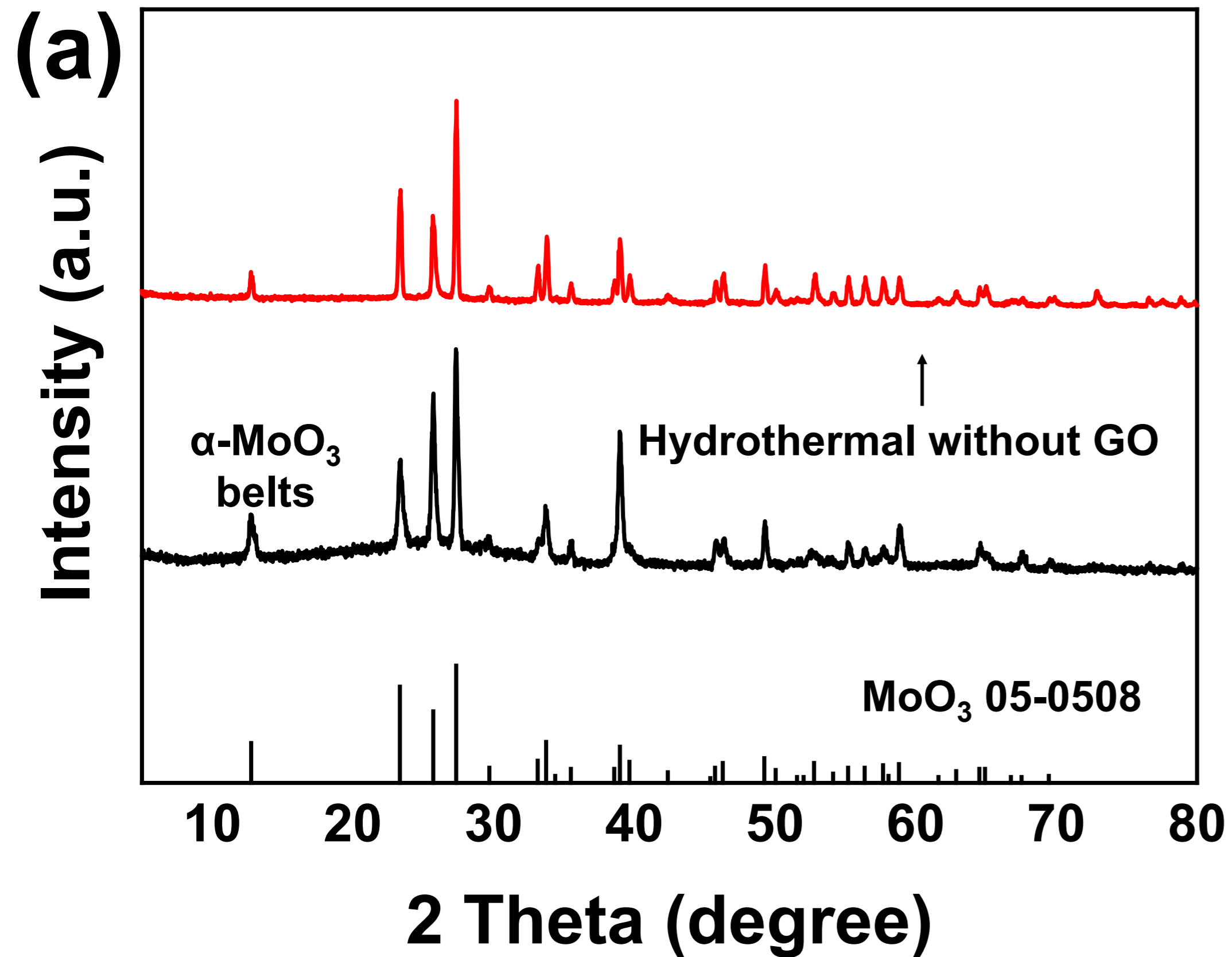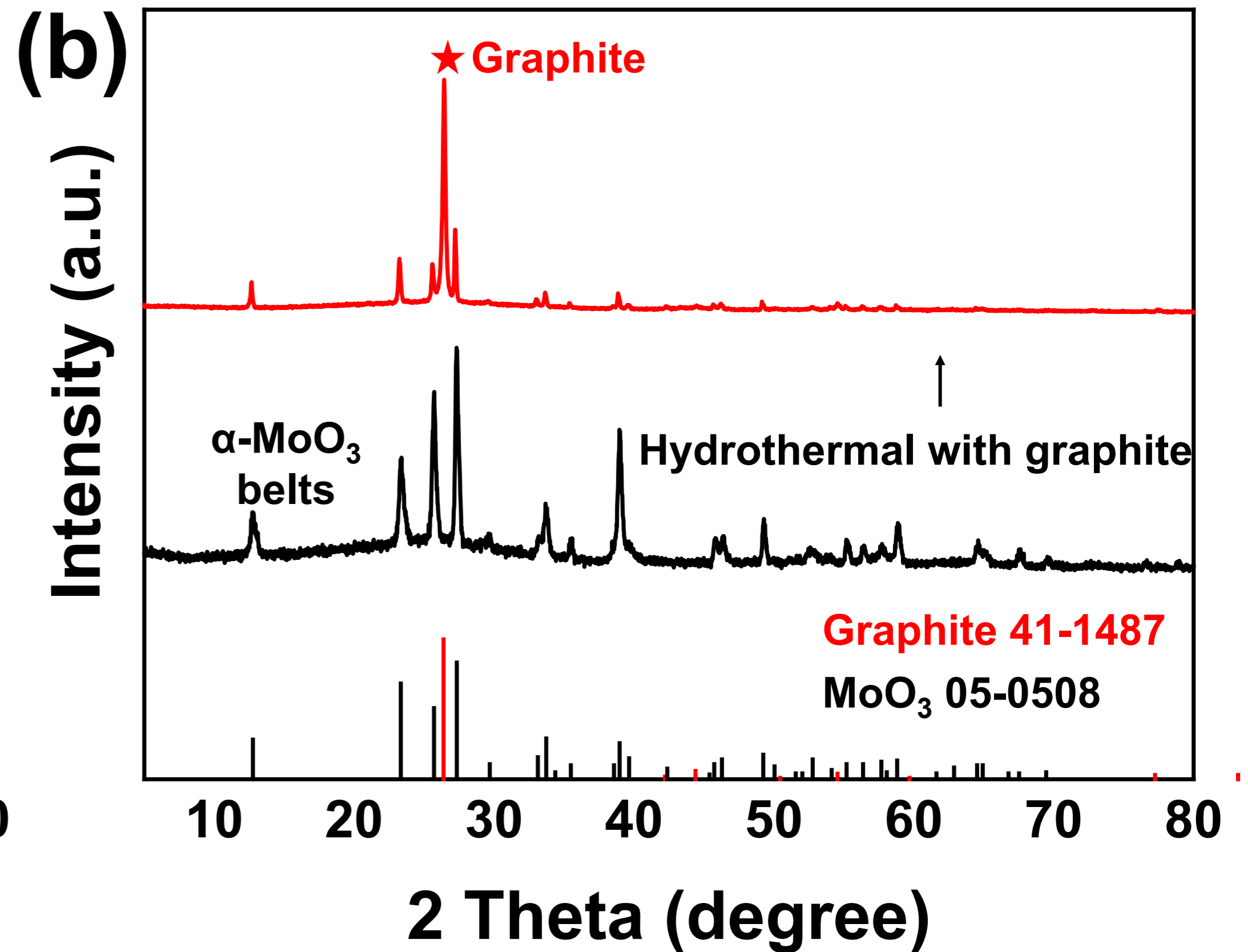

Supplement: Supplementary Materials — Figure S1: morphology characterization of α-MoO3 belts. Figure S2: crystal interlayer spacing analysis of α-MoO3 belts. Figure S3: crystal structure characterizations of α-MoO3 belts and the obtained A-MoO3-x/rGO hybrid fiber. Figure S4: crystal structure characterizations of hydrothermal treated α-MoO3 belts with different conditions. Figure S5: morphology characterizations of A-MoO3-x/rGO hybrid fibers obtained at different synthetic time. Figure S6: crystal structure characterizations of A-MoO3-x/rGO hybrid fibers obtained at different synthetic time. Figure S7: electrochemical properties of A-MoO3-x/rGO hybrid fibers obtained at different synthetic conditions. Figure S8: CV profiles of the pristine α-MoO3 belts. Figure S9: electrochemical properties of the optimized A-MoO3-x/rGO hybrid fiber. Figure S10: Nyquist plots of bare rGO fiber and A-MoO3-x/rGO hybrid fibers, respectively. Figure S11: analysis of capacitance contribution of optimized A-MoO3-x/rGO hybrid fiber. Figure S12: schematic illustration of the ion transport channels within α-MoO3 crystals and A-MoO3-x, respectively. [file 6742715.f1.zip › Yu_Figure of SI_Figure S4.pdf]

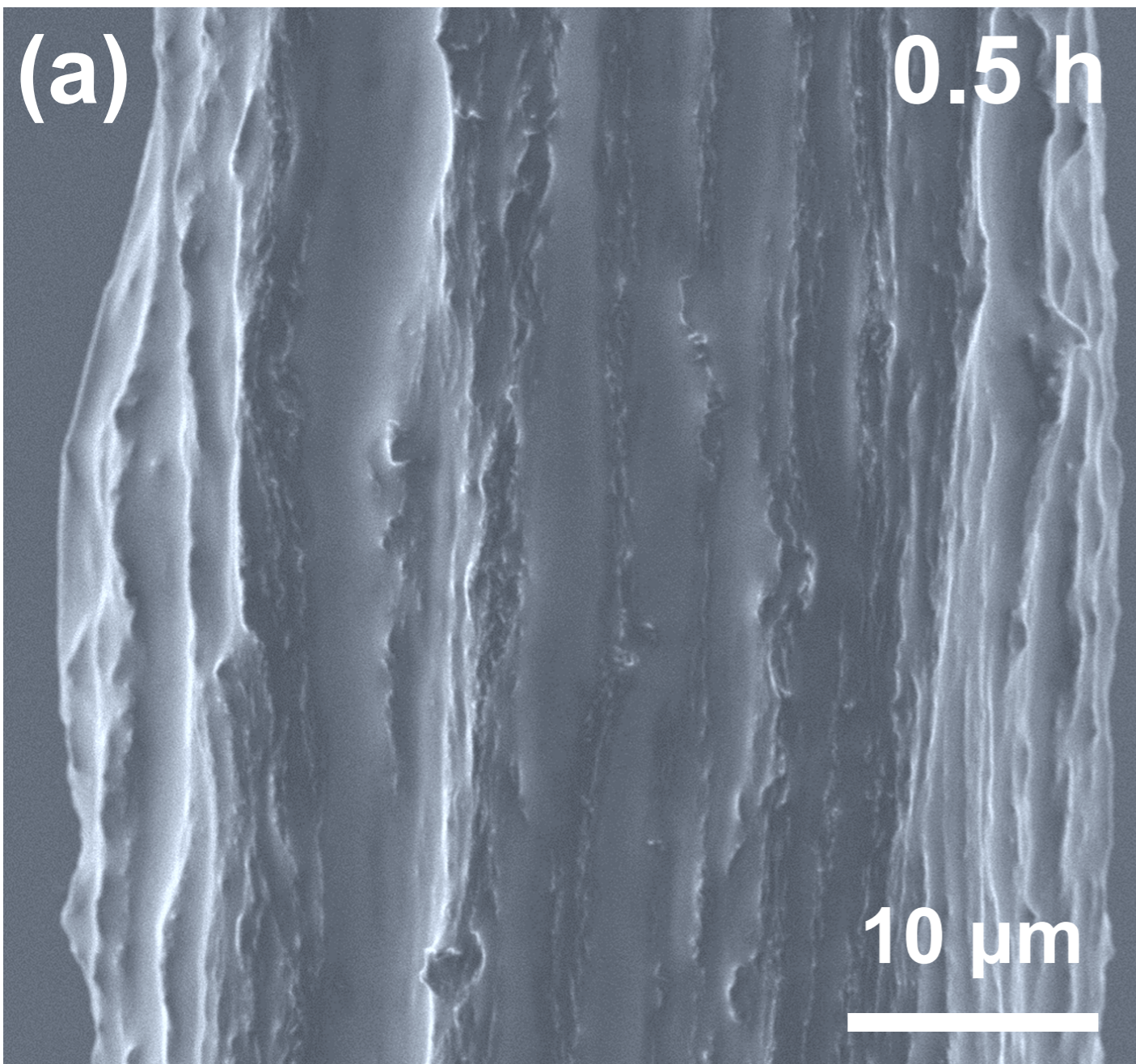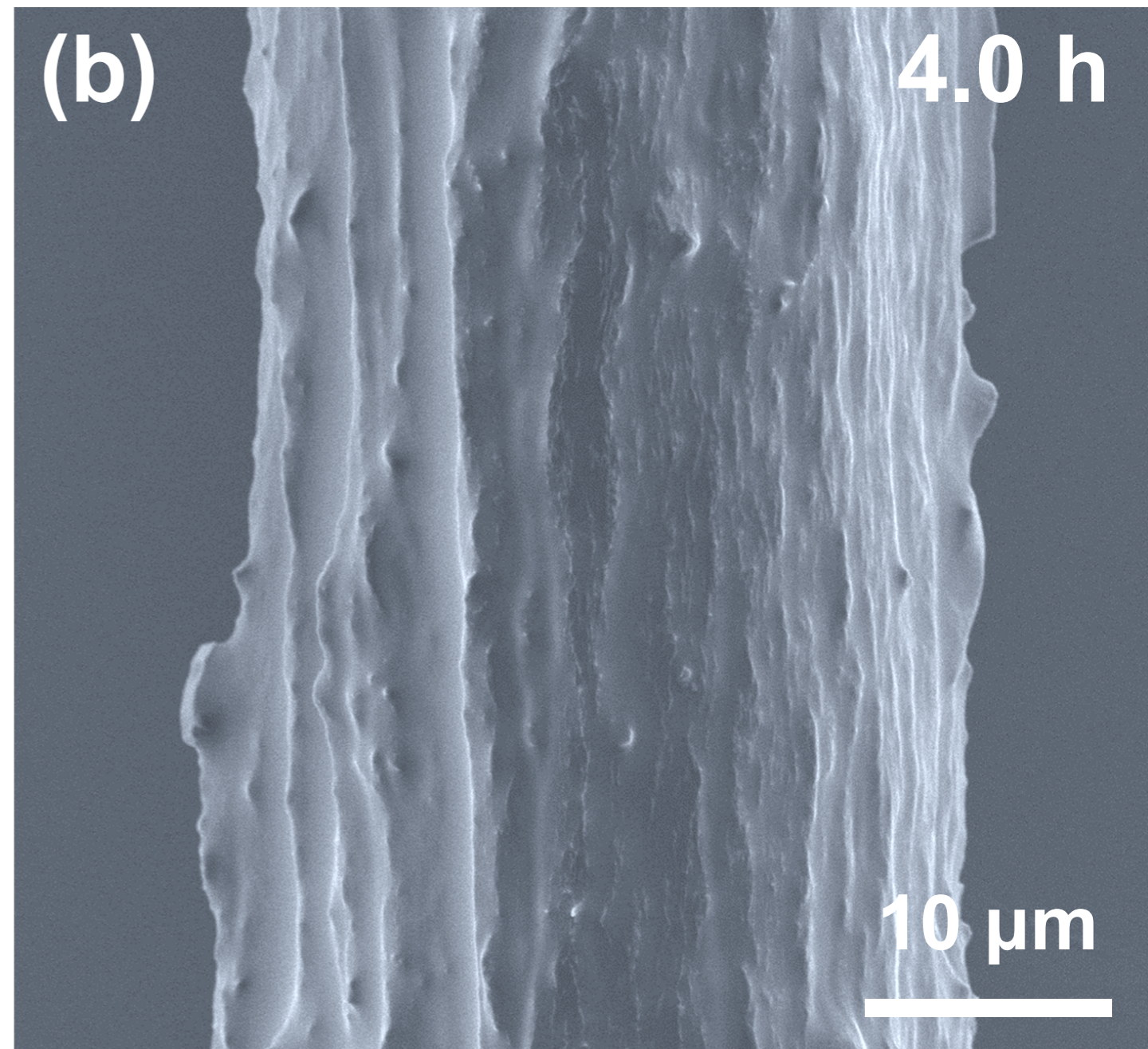

Supplement: Supplementary Materials — Figure S1: morphology characterization of α-MoO3 belts. Figure S2: crystal interlayer spacing analysis of α-MoO3 belts. Figure S3: crystal structure characterizations of α-MoO3 belts and the obtained A-MoO3-x/rGO hybrid fiber. Figure S4: crystal structure characterizations of hydrothermal treated α-MoO3 belts with different conditions. Figure S5: morphology characterizations of A-MoO3-x/rGO hybrid fibers obtained at different synthetic time. Figure S6: crystal structure characterizations of A-MoO3-x/rGO hybrid fibers obtained at different synthetic time. Figure S7: electrochemical properties of A-MoO3-x/rGO hybrid fibers obtained at different synthetic conditions. Figure S8: CV profiles of the pristine α-MoO3 belts. Figure S9: electrochemical properties of the optimized A-MoO3-x/rGO hybrid fiber. Figure S10: Nyquist plots of bare rGO fiber and A-MoO3-x/rGO hybrid fibers, respectively. Figure S11: analysis of capacitance contribution of optimized A-MoO3-x/rGO hybrid fiber. Figure S12: schematic illustration of the ion transport channels within α-MoO3 crystals and A-MoO3-x, respectively. [file 6742715.f1.zip › Yu_Figure of SI_Figure S5.pdf]

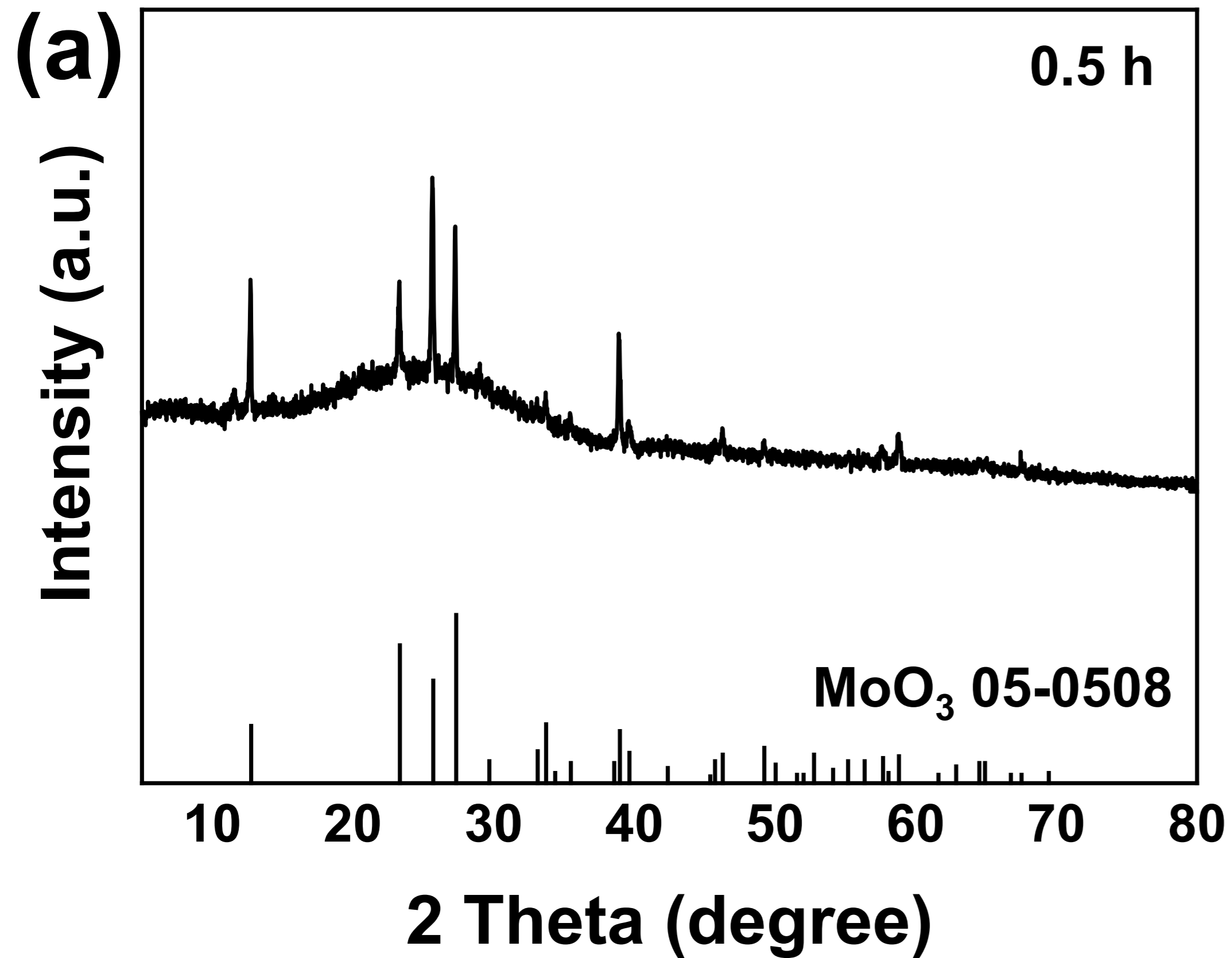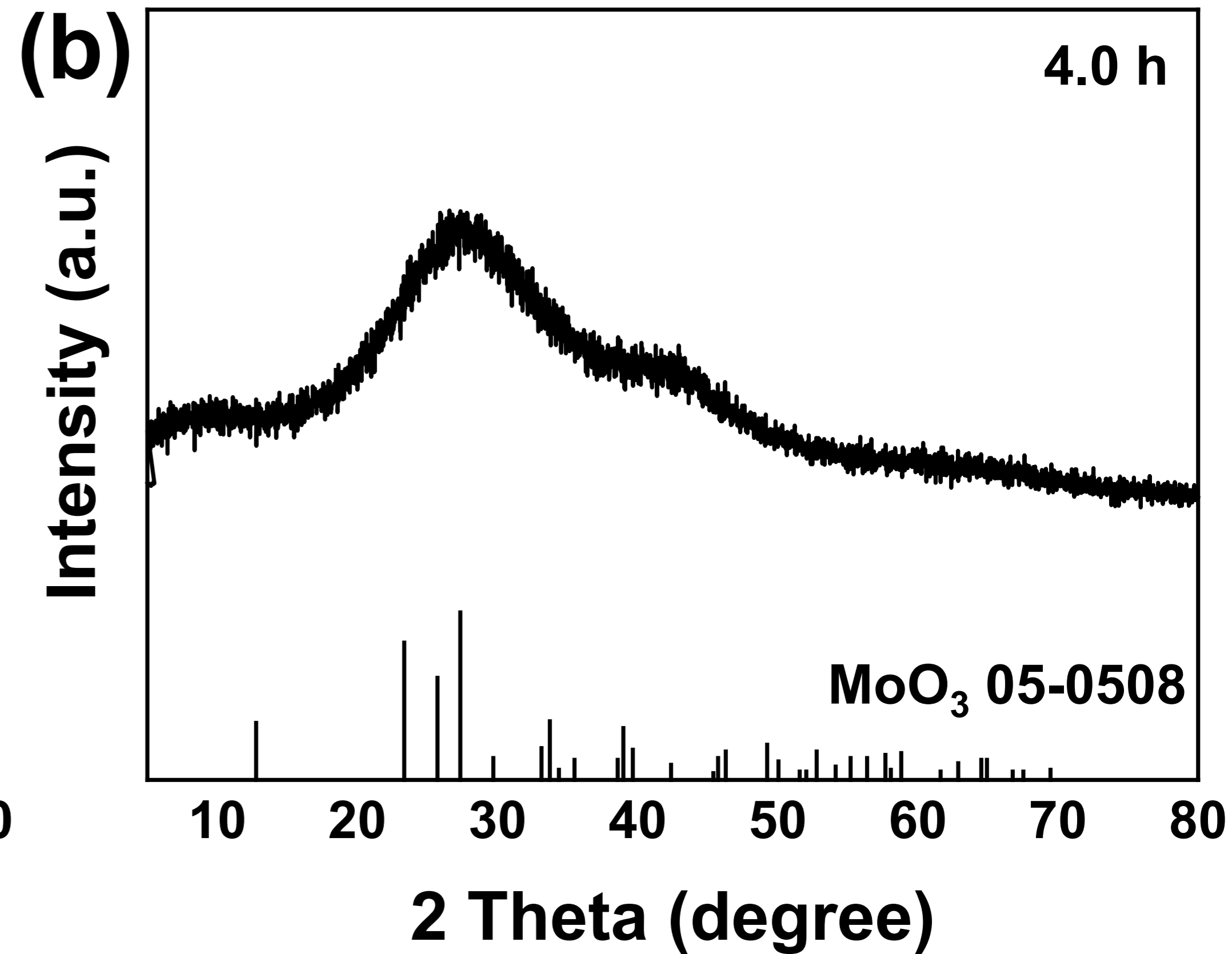

Supplement: Supplementary Materials — Figure S1: morphology characterization of α-MoO3 belts. Figure S2: crystal interlayer spacing analysis of α-MoO3 belts. Figure S3: crystal structure characterizations of α-MoO3 belts and the obtained A-MoO3-x/rGO hybrid fiber. Figure S4: crystal structure characterizations of hydrothermal treated α-MoO3 belts with different conditions. Figure S5: morphology characterizations of A-MoO3-x/rGO hybrid fibers obtained at different synthetic time. Figure S6: crystal structure characterizations of A-MoO3-x/rGO hybrid fibers obtained at different synthetic time. Figure S7: electrochemical properties of A-MoO3-x/rGO hybrid fibers obtained at different synthetic conditions. Figure S8: CV profiles of the pristine α-MoO3 belts. Figure S9: electrochemical properties of the optimized A-MoO3-x/rGO hybrid fiber. Figure S10: Nyquist plots of bare rGO fiber and A-MoO3-x/rGO hybrid fibers, respectively. Figure S11: analysis of capacitance contribution of optimized A-MoO3-x/rGO hybrid fiber. Figure S12: schematic illustration of the ion transport channels within α-MoO3 crystals and A-MoO3-x, respectively. [file 6742715.f1.zip › Yu_Figure of SI_Figure S6.pdf]

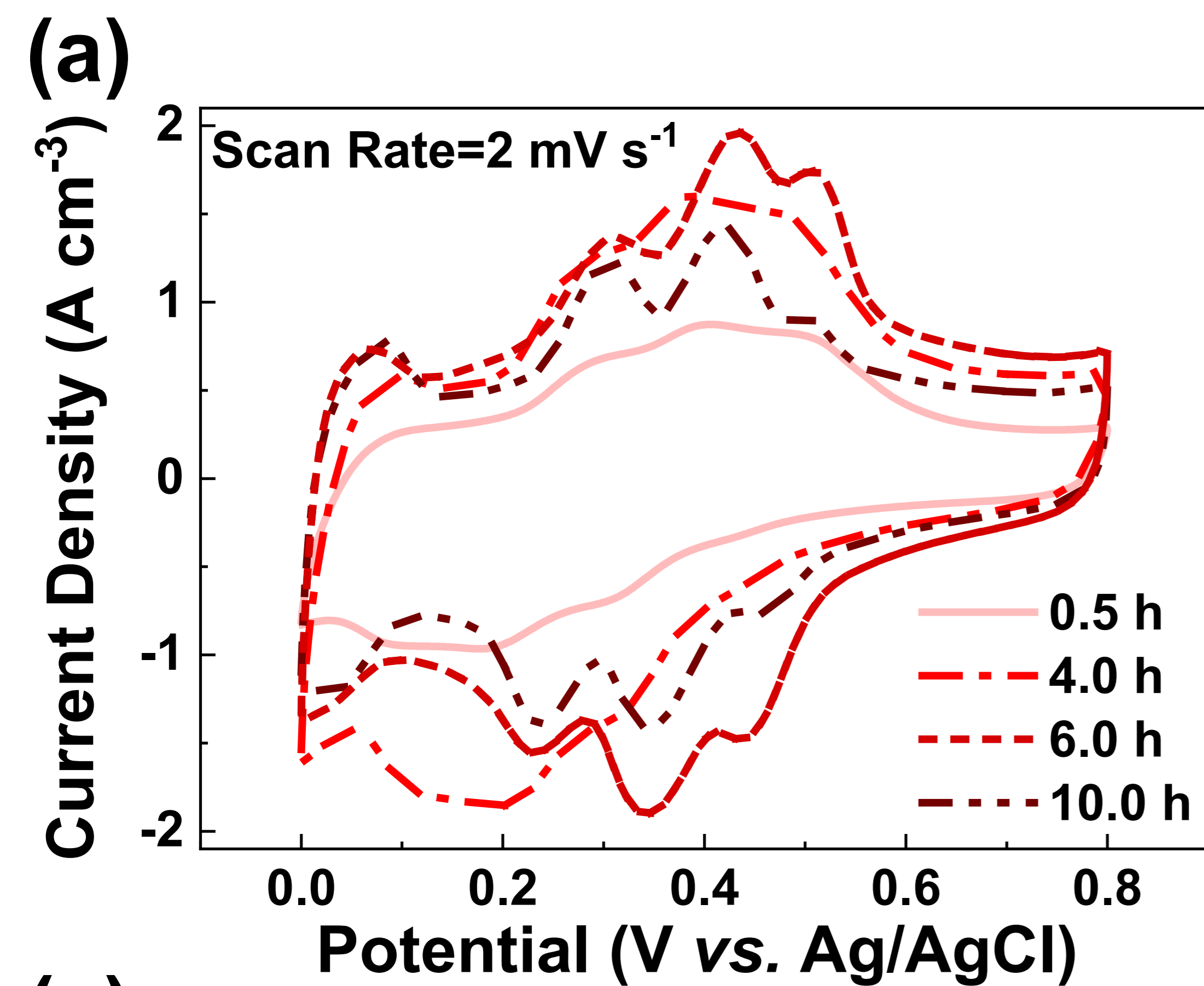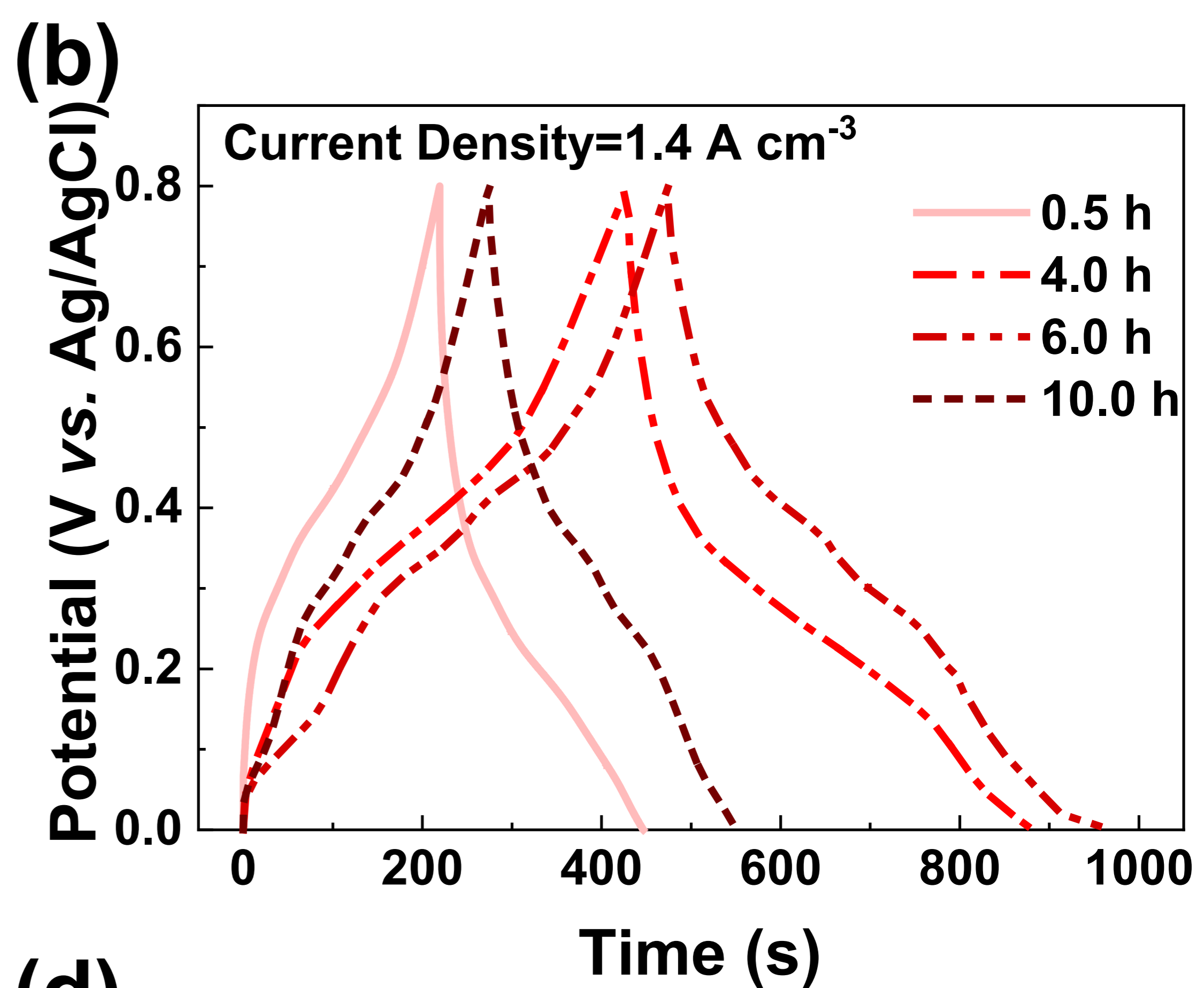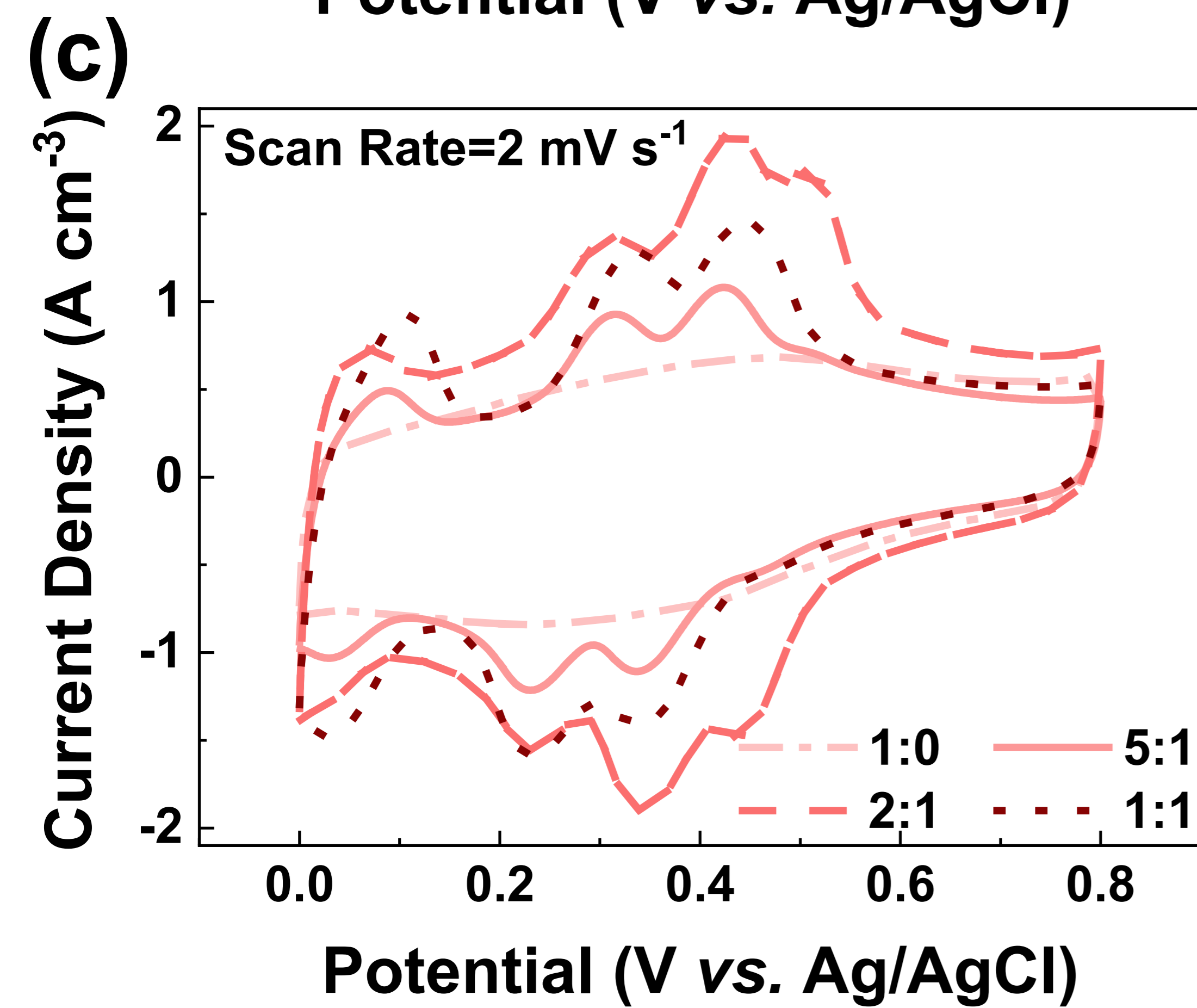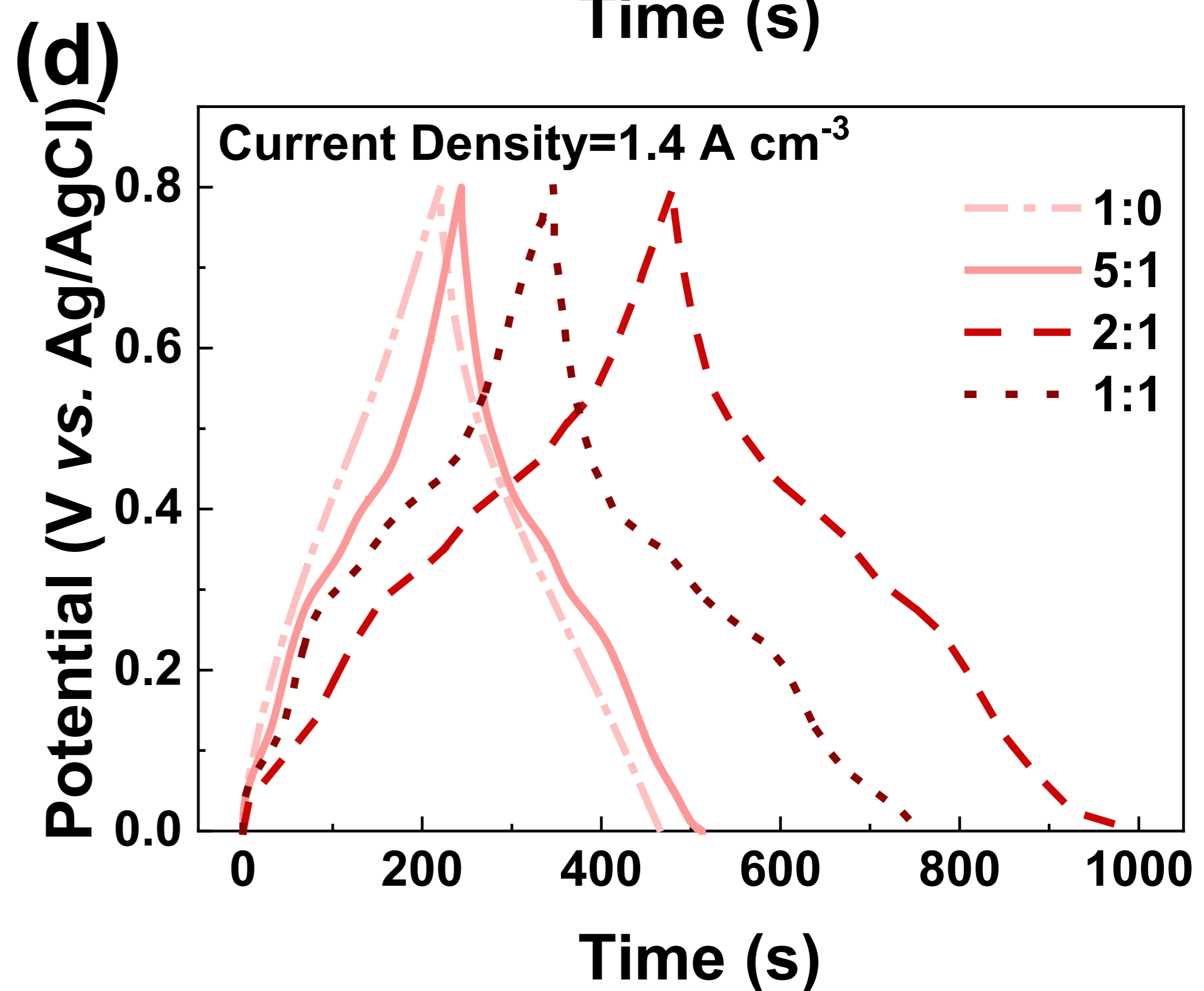

Supplement: Supplementary Materials — Figure S1: morphology characterization of α-MoO3 belts. Figure S2: crystal interlayer spacing analysis of α-MoO3 belts. Figure S3: crystal structure characterizations of α-MoO3 belts and the obtained A-MoO3-x/rGO hybrid fiber. Figure S4: crystal structure characterizations of hydrothermal treated α-MoO3 belts with different conditions. Figure S5: morphology characterizations of A-MoO3-x/rGO hybrid fibers obtained at different synthetic time. Figure S6: crystal structure characterizations of A-MoO3-x/rGO hybrid fibers obtained at different synthetic time. Figure S7: electrochemical properties of A-MoO3-x/rGO hybrid fibers obtained at different synthetic conditions. Figure S8: CV profiles of the pristine α-MoO3 belts. Figure S9: electrochemical properties of the optimized A-MoO3-x/rGO hybrid fiber. Figure S10: Nyquist plots of bare rGO fiber and A-MoO3-x/rGO hybrid fibers, respectively. Figure S11: analysis of capacitance contribution of optimized A-MoO3-x/rGO hybrid fiber. Figure S12: schematic illustration of the ion transport channels within α-MoO3 crystals and A-MoO3-x, respectively. [file 6742715.f1.zip › Yu_Figure of SI_Figure S7.pdf]

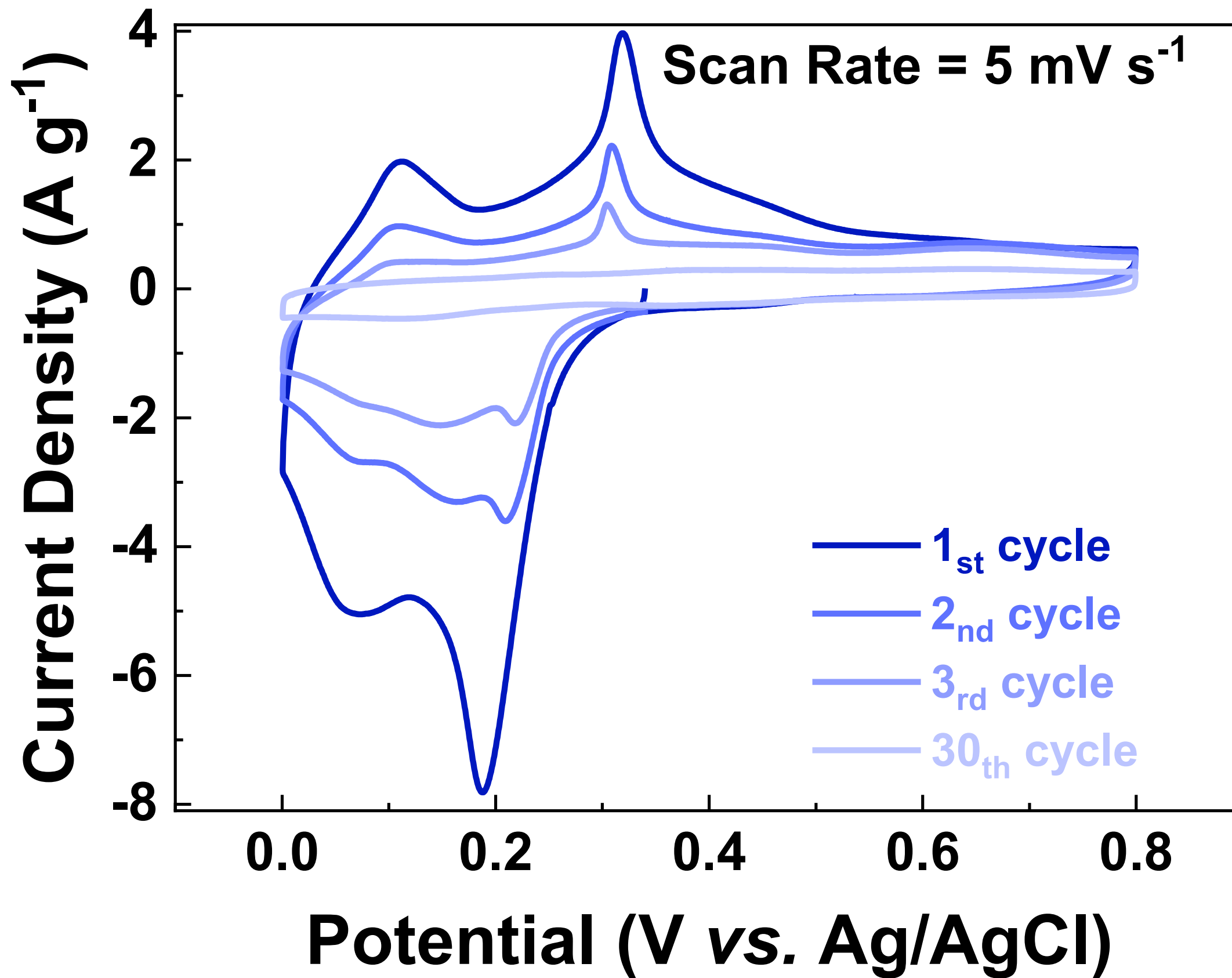

Supplement: Supplementary Materials — Figure S1: morphology characterization of α-MoO3 belts. Figure S2: crystal interlayer spacing analysis of α-MoO3 belts. Figure S3: crystal structure characterizations of α-MoO3 belts and the obtained A-MoO3-x/rGO hybrid fiber. Figure S4: crystal structure characterizations of hydrothermal treated α-MoO3 belts with different conditions. Figure S5: morphology characterizations of A-MoO3-x/rGO hybrid fibers obtained at different synthetic time. Figure S6: crystal structure characterizations of A-MoO3-x/rGO hybrid fibers obtained at different synthetic time. Figure S7: electrochemical properties of A-MoO3-x/rGO hybrid fibers obtained at different synthetic conditions. Figure S8: CV profiles of the pristine α-MoO3 belts. Figure S9: electrochemical properties of the optimized A-MoO3-x/rGO hybrid fiber. Figure S10: Nyquist plots of bare rGO fiber and A-MoO3-x/rGO hybrid fibers, respectively. Figure S11: analysis of capacitance contribution of optimized A-MoO3-x/rGO hybrid fiber. Figure S12: schematic illustration of the ion transport channels within α-MoO3 crystals and A-MoO3-x, respectively. [file 6742715.f1.zip › Yu_Figure of SI_Figure S8.pdf]

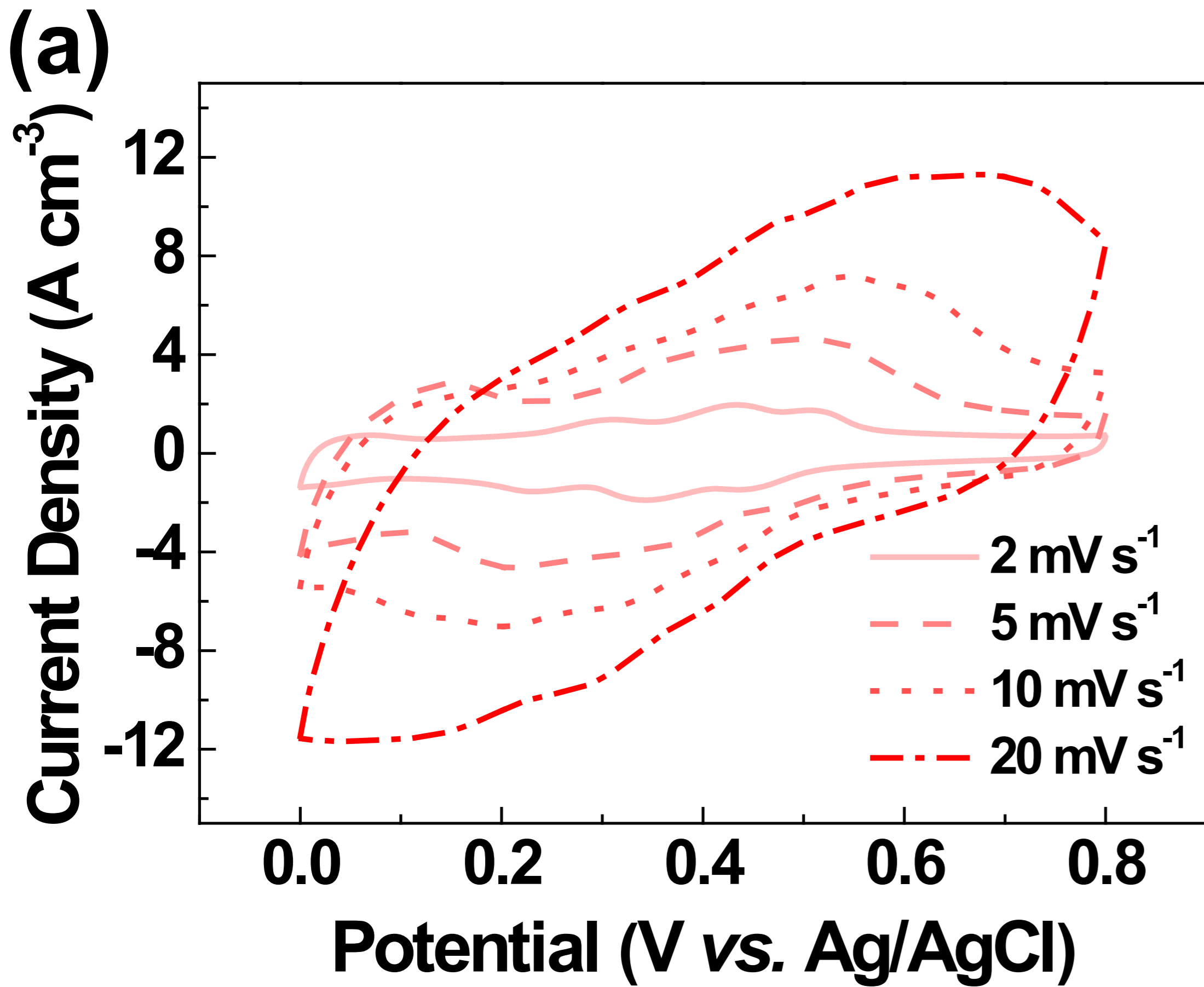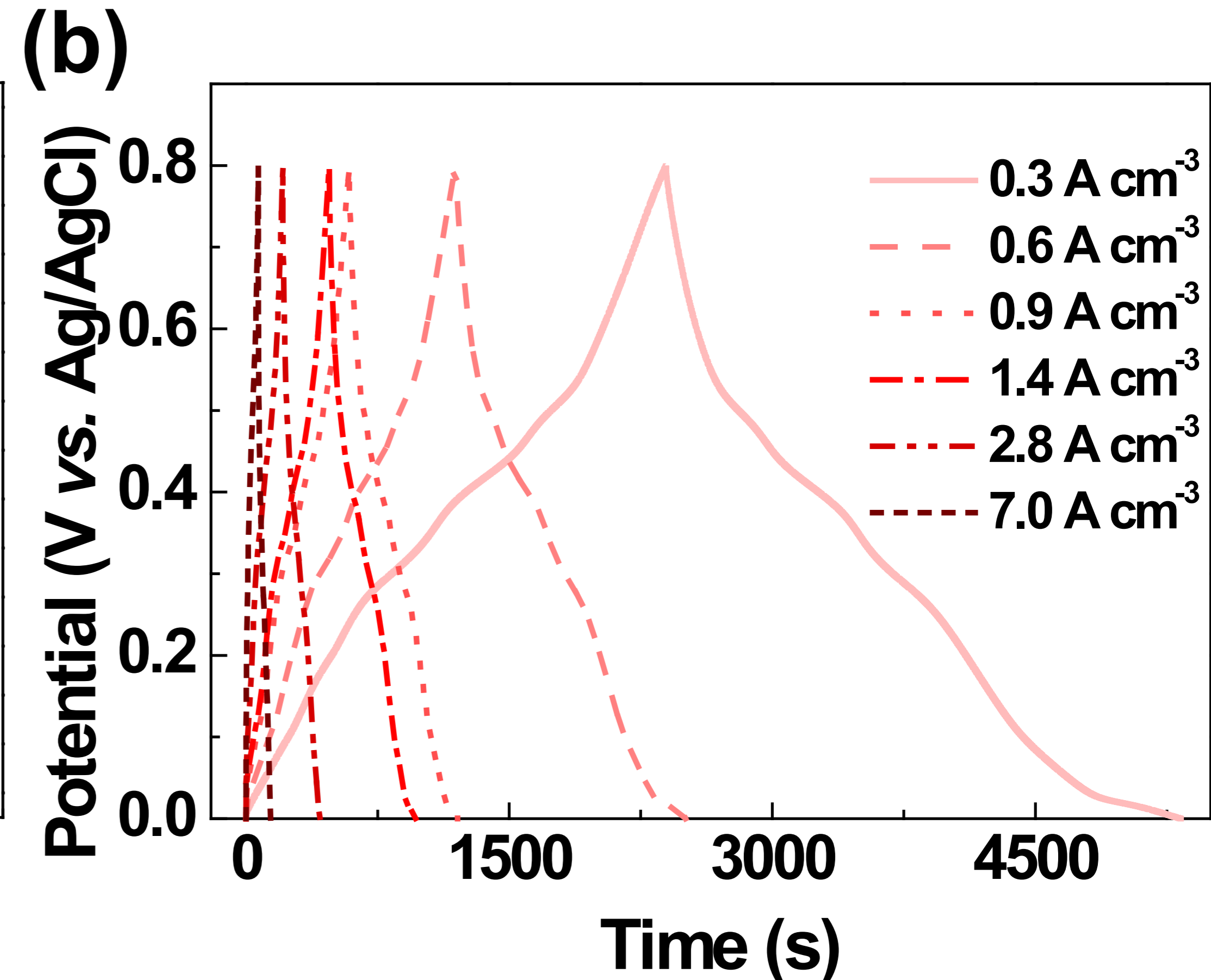

Supplement: Supplementary Materials — Figure S1: morphology characterization of α-MoO3 belts. Figure S2: crystal interlayer spacing analysis of α-MoO3 belts. Figure S3: crystal structure characterizations of α-MoO3 belts and the obtained A-MoO3-x/rGO hybrid fiber. Figure S4: crystal structure characterizations of hydrothermal treated α-MoO3 belts with different conditions. Figure S5: morphology characterizations of A-MoO3-x/rGO hybrid fibers obtained at different synthetic time. Figure S6: crystal structure characterizations of A-MoO3-x/rGO hybrid fibers obtained at different synthetic time. Figure S7: electrochemical properties of A-MoO3-x/rGO hybrid fibers obtained at different synthetic conditions. Figure S8: CV profiles of the pristine α-MoO3 belts. Figure S9: electrochemical properties of the optimized A-MoO3-x/rGO hybrid fiber. Figure S10: Nyquist plots of bare rGO fiber and A-MoO3-x/rGO hybrid fibers, respectively. Figure S11: analysis of capacitance contribution of optimized A-MoO3-x/rGO hybrid fiber. Figure S12: schematic illustration of the ion transport channels within α-MoO3 crystals and A-MoO3-x, respectively. [file 6742715.f1.zip › Yu_Figure of SI_Figure S9.pdf]
